# Small contribution of gold mines to the ongoing tuberculosis epidemic in South Africa: a modeling-based study

**DOI:** 10.1186/s12916-018-1037-3

**Published:** 2018-04-12

**Authors:** Stewart T. Chang, Violet N. Chihota, Katherine L. Fielding, Alison D. Grant, Rein M. Houben, Richard G. White, Gavin J. Churchyard, Philip A. Eckhoff, Bradley G. Wagner

**Affiliations:** 1 Institute for Disease Modeling, Bellevue, Washington USA; 2 Aurum Institute, Johannesburg, South Africa; 3 School of Public Health, Faculty of Health Sciences, University of Witwatersrand, Johannesburg, South Africa; 4 Foundation for Innovative New Diagnostics, Geneva, Switzerland; 5 Department of Infectious Disease Epidemiology, London School of Hygiene and Tropical Medicine, London, UK; 6 Department of Clinical Research, London School of Hygiene and Tropical Medicine, London, UK; 7 Africa Health Research Institute, School of Nursing and Public Health, University of KwaZulu-Natal, Durban, South Africa; 8 TB Modelling Group, CMMID, TB Centre, London School of Hygiene and Tropical Medicine, London, UK; 9 Advancing Treatment and Care for TB/HIV, South African Medical Research Council, Johannesburg, South Africa

**Keywords:** Tuberculosis, HIV, Mining, South Africa, Risk groups, Hotspots, Global health

## Abstract

**Background:**

Gold mines represent a potential hotspot for *Mycobacterium tuberculosis (Mtb)* transmission and may be exacerbating the tuberculosis (TB) epidemic in South Africa. However, the presence of multiple factors complicates estimation of the mining contribution to the TB burden in South Africa.

**Methods:**

We developed two models of TB in South Africa, a static risk model and an individual-based model that accounts for longer-term trends. Both models account for four populations — mine workers, peri-mining residents, labor-sending residents, and other residents of South Africa — including the size and prevalence of latent TB infection, active TB, and HIV of each population and mixing between populations. We calibrated to mine- and country-level data and used the static model to estimate force of infection (FOI) and new infections attributable to local residents in each community compared to other residents. Using the individual-based model, we simulated a counterfactual scenario to estimate the fraction of overall TB incidence in South Africa attributable to recent transmission in mines.

**Results:**

We estimated that the majority of FOI in each community is attributable to local residents: 93.9% (95% confidence interval 92.4–95.1%), 91.5% (91.4–91.5%), and 94.7% (94.7–94.7%) in gold mining, peri-mining, and labor-sending communities, respectively. Assuming a higher rate of *Mtb* transmission in mines, 4.1% (2.6–5.8%), 5.0% (4.5–5.5%), and 9.0% (8.8–9.1%) of new infections in South Africa are attributable to gold mine workers, peri-mining residents, and labor-sending residents, respectively. Therefore, mine workers with TB disease, who constitute ~ 2.5% of the prevalent TB cases in South Africa, contribute 1.62 (1.04–2.30) times as many new infections as TB cases in South Africa on average. By modeling TB on a longer time scale, we estimate 63.0% (58.5–67.7%) of incident TB disease in gold mining communities to be attributable to recent transmission, of which 92.5% (92.1–92.9%) is attributable to local transmission.

**Conclusions:**

Gold mine workers are estimated to contribute a disproportionately large number of *Mtb* infections in South Africa on a per-capita basis. However, mine workers contribute only a small fraction of overall *Mtb* infections in South Africa. Our results suggest that curtailing transmission in mines may have limited impact at the country level, despite potentially significant impact at the mining level.

**Electronic supplementary material:**

The online version of this article (10.1186/s12916-018-1037-3) contains supplementary material, which is available to authorized users.

## Background

Gold mines in South Africa have historically been implicated in initiating the tuberculosis (TB) epidemic in South Africa. As Packard notes, “The immense size of the mine labor force, over 200,000 on the Rand alone by 1910, together with the appalling health conditions that existed on the mines, ensured that they would play a central role in the early development of TB in southern Africa” [1].

To what extent gold mines continue to contribute to TB in South Africa, however, is subject to debate. Several factors complicate this question. Within the mines, crowding, insufficient ventilation, and warm, humid air may increase the rate of *Mycobacterium tuberculosis (Mtb)* transmission. Biological and social factors may then affect the extent to which *Mtb* spreads among mine workers and from mine workers to other groups. For example, mine workers and residents of other areas with whom they interact may already have latent tuberculosis infection (LTBI), which confers partial immunity to reinfection despite posing a longer-term risk for reactivation in the future [2]. Both mine workers and residents of other communities may also carry high burdens of HIV infection, increasing their rate of reactivation [3–5]. Finally, mixing patterns between mine workers and other residents may determine to what extent mine workers contribute to the larger epidemic [2, 6]. For example, estimating the risk of TB infection in peri-mining residents due to mine workers requires one to account for the probability that susceptible peri-mining residents come into contact with infectious mine workers, which depends on the size of each group, the prevalence of LTBI and active TB in each group, and the amount of time the groups spend together. On a longer time scale, labor-related migration and repatriation of mine workers are also likely to affect how widely mine workers may spread infections [2, 6].

Mathematical models have served as useful tools for understanding the TB epidemic in South Africa. For example, at the country level, models have been used to predict the impact of implementing different interventions [7–10]. Models have also proved useful for understanding more local disease dynamics, e.g., at the level of a city [11] or in specific environments such as a prison [12] or a household [13]. More recently, models have also been applied to the gold mines in South Africa to understand the results of the Thibela TB study, which tested a sustained campaign of preventive therapy among mine workers [14, 15]. However, models have not yet addressed how mine workers mix with other groups and whether these interactions contribute to overall TB burden.

To estimate the contribution of gold mines to the ongoing TB epidemic in South Africa, we developed two computational models and applied them to gold mine workers and mining-related groups in South Africa. First, we developed a simplified static risk model that accounts for data on gold mine workers and peri-mining, labor-sending, and other residents of South Africa and estimates the force of infection (FOI) and fraction of transmission events (new infections) in each community that are attributable to local residents compared to residents from other areas. Secondly, we developed a dynamic, individual-based model of TB that accounts for longer-term trends in demographics and risk factors and also features a more detailed disease natural history to estimate the fraction of incidence attributable to transmission from gold mine workers. Together, these tools provide quantitative estimates that address to what extent gold mines are continuing to contribute to the TB epidemic in South Africa.

## Methods

### Epidemiological data sources

We consider four residency groups in South Africa: gold mine workers, peri-mining residents, labor-sending residents, and other residents of South Africa. Our primary data source for mine workers was data collected during the Thibela TB study [16]. Peri-mining communities were identified based on proximity to Thibela TB study sites, comprising the Lejweleputswa (Free State province), West Rand (Gauteng province), and Dr Kenneth Kaunda (North West province) districts. Labor-sending communities were identified as the OR Tambo and Alfred Nzo (Eastern Cape province) and Ugu and Sisonke (KwaZulu-Natal province) districts. Other residents of South Africa were assumed to comprise all remaining districts. Residents of areas outside of South Africa were not considered.

### Input parameters

In the models we accounted for three general categories of parameters: population size, disease natural history, and population mixing. Population sizes were taken from the South Africa Census 2011 [17]. For the mine worker population, we considered both gold mine workers and mine workers of other commodities, representing an upper limit on the at-risk population [18, 19]. The epidemiological characteristics of each population were derived from the literature. Measurements of TB incidence and prevalence at the country level were taken from World Health Organization estimates [20] and at the mine level from the Thibela TB study [21], while HIV prevalence and antiretroviral therapy (ART) coverage were taken from Joint United Nations Programme on HIV/AIDS (UNAIDS)-based measurements [22]. Disease natural history parameters were similar to those found in other TB models and included the rate at which infected individuals progress to active disease as a result of primary disease or reactivation, the effect of HIV on reactivation, and the frequency of different forms of active disease (smear-positive, smear-negative, and extra-pulmonary) and relative infectiousness of each form (Additional file 1: Table S1). Parameters specific to mining included a multiplier for increased *Mtb* transmission in the mines, the prevalence of silicosis among mine workers, and the effect of silicosis on reactivation (Additional file 1: Table S2). South Africa-specific estimates of healthcare access and treatment effectiveness via directly observed therapy short-course (DOTS) were also included (Additional file 1: Table S3). Population mixing parameters were taken from national tourism surveys [23] as well as data collected during the Thibela TB study (Additional file 1: Table S4).

### Description of static risk model (spreadsheet model)

To account for the current state of the TB epidemic in mining and mining-related communities in South Africa and short-term, sub-annum processes that relate to TB transmission between these communities, we developed a static risk model. The model represents a Taylor series-type approximation of dynamic processes such as the generation of new infections given the number of susceptibles and prevalent cases in each population and the effect of risk factors such as increased *Mtb* transmission in the mines and HIV in the overall population. These quantities are not updated iteratively in the model; therefore, the model represents a short-term, 1-year projection of these quantities.

**Fig. 1:**
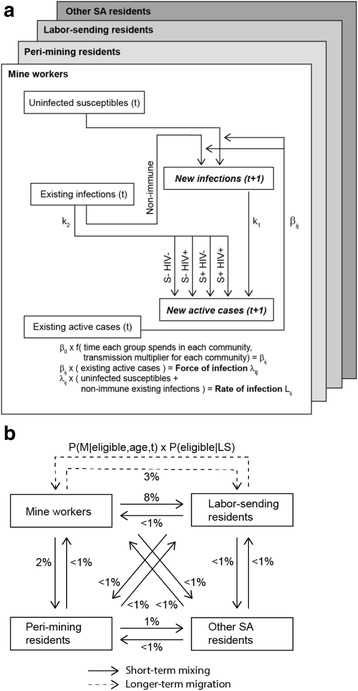
Disease state transitions and groups represented in the static risk and individual-based models. **a** Disease transitions in the static risk model were limited to new infections and reactivation from existing latent infections, while the individual-based model also represented longer-term processes such as new infections contributing to prevalence (not shown). **b** Population mixing patterns as fraction of time per annum spent in a different community (for short-term mixing) or probability of residency change per annum (for longer-term migration). Both static risk and individual-based models represent short-term mixing, but only the individual-based model represents longer-term migration. *S+/−* silicosis presence/absence, *M* mining resident, *LS* labor-sending resident

The static risk model (spreadsheet model) was encoded in Excel and comprises formulas to calculate the FOI (per-susceptible rate of infection) and number of infections occurring in each group (Fig. 1a). A “who acquires infection from whom” (WAIFW) matrix [24] was derived where each element *β*_ij_ represents the rate of *Mtb* transmission from infectives in group *j* to susceptibles in group *i* for every community *k* where contact was assumed possible (spreadsheet matrix 1). Each element of the WAIFW matrix was based on a base rate of transmission *β*_0_ which we defined as the number of new infections generated by each infective case per year averaged over smear-positive, smear-negative, and extra-pulmonary forms of disease. *β*_0_ was multiplied by a community-specific transmission multiplier *c*_k_, which accounts for multiple environmental factors and was calibrated in the individual-based model, and the fractions of each year *p*_ik_ and *p*_jk_ that individuals from groups *i* and *j*, respectively, spend in community *k* (Fig. 1b). *p*_ik_ and *p*_jk_ were converted to a frequency of contact between individuals from groups *i* and *j* in community *k* by dividing by the total number of individuals *N*_k_ present in community *k* at any given time, i.e., assuming frequency-dependent transmission [25]. *β*_ij_ was then calculated by summing this product over the set *A* of all communities *k* where groups *i* and *j* spend time.

$$ {\beta}_{ij}={\beta}_0{\sum}_{k\in A}\frac{c_k{p}_{ik}{p}_{jk}}{N_k} $$

Additional quantities were derived using the WAIFW matrix:

- *λ*_*ij*_: The FOI among susceptibles in group *i* attributable to infectives from group *j*, calculated by multiplying *β*_ij_ by the number of infectives in group *j* (spreadsheet matrix 2). The sum over all *j* for a given *i* provides the overall probability of infection per year for susceptibles in group *i*.
- *L*_ij_: The rate of infection in group *i* per-capita attributable to group *j*, calculated by multiplying *λ*_*ij*_ by the fraction of group *i* who are susceptible where susceptible is defined as uninfected or latently infected but susceptible to reinfection (spreadsheet matrix 3).
- *PAF(λ*_*ij*_*)*: The population attributable fraction (PAF) of FOI in group *i* attributable to group *j*, calculated by dividing each by the sum of *λ*_*ij*_ over all *j* for a given *i* (spreadsheet matrix 4).
- *PAF(L*_*j*_*)*: The PAF of all new infections in South Africa attributable to group *j*, calculated by dividing the number of infections attributable to group *j* (i.e., the sum of *L*_ij_ over all *i* for a given *j*) by the total number of infections (i.e., the sum of *L*_ij_ over all *i* and all *j*) (spreadsheet matrix 5).
- *pcPAF(L*_*j*_*)*: The PAF of all new infections in South Africa attributable to group *j* relative to the size of *j* (i.e., per-capita *j*) calculated by dividing *PAF(L*_*j*_*)* by the fraction of the South African population that group *j* represents (spreadsheet matrix 6). This represents the contribution of a particular group relative to its population size including both susceptible and infected individuals.

In the static risk model we also calculated a near-term estimate of TB incidence *I*_i_ in each group *i* (Fig. 1a). In this case near-term refers to 1 year in the future, as prevalence after the first year was not updated to include new cases or losses due to treatment or death. Near-term incidence accounted for TB cases from primary disease resulting from new infections in a given year and TB cases from reactivation of a stable pool of latent infections. Specifically near-term incidence was calculated as the sum of incidence from five sources: (1) primary disease from new infections (*k*_1_*L*_i_), (2) reactivation from non-silicotic, non-HIV-positive latent infections (*k*_2_(1 – *S*_i_)(1 – *H*_i_)*P*_i_), (3) reactivation from non-silicotic, HIV-positive latent infections (*k*_2_*m*_0,H_(1 – *S*_i_)*H*_i_*P*_i_), (4) reactivation from silicotic, non-HIV-positive latent infections (*k*_2_*m*_s,0_*S*_i_(1 – *H*_i_)*P*_i_), and (5) reactivation from silicotic, HIV-positive latent infections (*k*_2_*m*_s,H_*S*_i_*H*_i_*P*_i_). Here *k*_1_ and *k*_2_ represent base rates of primary disease in newly infected and reactivation in latently infected individuals, respectively, and *L*_i_ was derived from *L*_ij_ above. *S*_i_, *H*_i_, and *P*_i_ represent the stable prevalence of silicosis, HIV, and LTBI in group *i*, and *m*_s,0_, *m*_0,H_, and *m*_s,H_ represent multipliers on *k*_2_ for silicotic, HIV-positive, and simultaneously silicotic and HIV-positive individuals, respectively. HIV-positive latent infections were further subdivided into those receiving or not receiving ART, each assigned a separate multiplier on *k*_2_. Because the static risk model did not account for longer-term migration, e.g., for mine workers repatriating to labor-sending areas, the prevalence of silicotics in non-mining areas was assumed to be zero. The resulting values of *I*_i_ were compared to published values for mine workers from the Thibela TB study and for all South Africa from WHO estimates (spreadsheet matrix 7).

Monte Carlo simulations were performed where the mine-specific transmission rate and immunity from reinfection were sampled from normal distributions, while all other parameters were held at baseline values. For the mine-specific transmission rate, 95% of the density was assumed to lie within −20% and +20% of the baseline value. This range was selected based on the Gammaitoni-Nucci equation [26], which specifies that the probability of an individual acquiring an infection in a confined space increases exponentially as the inverse of ventilation rate. Therefore, a −20% or +20% difference in transmission rate could result from a +25% or −17% change in ventilation rate, respectively; these were similar to values from the individual-based model calibration (cf. Additional file 1: Figure S2B, S2C). For immunity from reinfection, 95% of the density was assumed to lie within −40% and +40% of the baseline value. One thousand randomly drawn pairs of values for these two parameters were used, and 95% confidence intervals (CIs) were taken from the 0.025 and 0.975 quantiles of the resulting output values. Both the static risk model and the individual-based model as well as input parameter files are available on GitHub (https://github.com/SCTX/mining_contribution).

### Description of individual-based model

While the static risk model accounts for the current state of the TB epidemic and processes occurring on a sub-annum time scale, particularly population mixing, it does not update the prevalence of different disease states iteratively and does not represent longer-term changes in demographics and risk factors such as HIV. To provide a longer-term representation of the TB epidemic, we developed a dynamic, individual-based model; this model was coded in C++ and based on the TB model available in the EMOD software package [27]. We briefly describe the model here; parameter values and additional details are provided in Additional file 1: Tables S1–S4. Individuals were assigned one of the following disease states: susceptible, latently infected, active pre-symptomatic, active symptomatic, and recovered. Individuals transitioned between these states randomly according to exponentially distributed delays. Birth and non-disease death processes were represented whereby individuals were added to and removed from the simulation at rates consistent for South Africa. A residency status in one of the four groups in the model (mining, peri-mining, labor-sending, and other South Africa) was assigned at birth. Residency status was retained for the lifetime of the individual except for individuals born with labor-sending group status who transitioned to mining status during adulthood and then back to labor-sending status upon retirement. Short-term mixing between groups representing regular visits from mine workers to peri-mining or labor-sending communities was specified by an interaction matrix as in the static risk model. The numbers of discrete agents in the model were scaled in the output to reflect the sizes of the real populations: individuals in mining, peri-mining, and labor-sending groups by approximately 50:1 and in other South Africa by approximately 400:1. These scale factors corresponded to 2011 population sizes of approximately 0.5 million (M), 2.1 M, and 3.4 M in mining, peri-mining, and labor-sending areas, respectively, and 47 M in the remainder of South Africa [17].

Two risk factors were represented in the individual-based model, HIV infection and silicosis. HIV infection was distributed to individuals according to age-specific rates of infection; these were generated from the EMOD HIV model calibrated to South Africa [28] and assumed to be the same across residency groups [29–31]. The EMOD HIV model assigned a CD4 count to each individual which declined linearly with time in the absence of treatment [29–31]. ART was distributed to HIV-positive individuals according to eligibility guidelines in South Africa and matched population coverage estimates [22]. ART had the effect of increasing CD4 levels in the model [29–31]. Silicosis was acquired by mining group individuals at a rate consistent with radiographic observations in mine workers, at approximately 1% per year of employment [32–34] (Additional file 1: Table S2).

Susceptible individuals in the model were infected at a rate that differed by residency group and depended on the total infectiousness of other groups and the frequency of group interactions. The infectiousness of each group depended on the prevalence of different forms of active TB, where pre-symptomatic, smear-negative, and extra-pulmonary forms of disease were assumed to contribute less infectiousness than smear-positive disease. Data on the frequency of group interactions were specified by a WAIFW matrix as in the static risk model [35].

Infected individuals transitioned to active disease with one of two rates representing primary and reactivation disease. Active disease in the model included a pre-symptomatic period of set duration followed by symptomatic disease of smear-positive, smear-negative, or extra-pulmonary forms. Individuals persisted in symptomatic disease until progressing to self-cure, treatment, or death. HIV and silicosis had the effect of increasing the rate of reactivation, represented as multipliers on the base rate of reactivation [36]. For HIV-positive individuals, the magnitude of the increase varied as the inverse of CD4 level.

Individuals with active symptomatic disease were assumed to seek care at high or low rates corresponding to high- or low-quality access to care, respectively, broadly representing different levels of care in South Africa. Upon accessing care, symptomatic individuals were assumed to receive a sputum smear or GeneXpert test, depending on whether care was sought before or during DOTS availability. The probability of a positive test result corresponded to observed test sensitivities. If a positive test result was obtained, an individual was assumed to undergo treatment, with a rate of disease clearance that depended on whether treatment was given before or during DOTS availability. Following treatment, individuals transitioned to a recovered state that was identical to the susceptible state but assumed to have a reduced probability of reinfection due to immunity.

### Individual-based model calibration and application

Several historical population-wide events were simulated in the individual-based model. During each simulation the model was seeded and run for a specified burn-in period. With a burn-in period of 100 simulated years, incidence and mortality were observed to be stable in the different groups in the model, consistent with endemic TB. HIV, DOTS, and ART were introduced at simulated years 1985, 2002, and 2007, respectively, representing country-wide trends.

The model was calibrated to TB incidence in mining areas measured during the Thibela TB study [21] and TB incidence and mortality at the country level for multiple years [37]. Parameters for the transmission rate in mining areas and immunity to reinfection following previous TB exposure were varied during calibration. The transmission rate in mining areas was parameterized as a multiple of the base transmission rate and represented the aggregate environmental factors, e.g., reduced ventilation rates, that may increase *Mtb* transmission in mines. A likelihood score for each parameter combination was computed using a likelihood function based on a normal distribution where the differences between published high and low estimates of incidence and mortality were taken to represent 95% CIs and the three epidemiological indicators were equally weighted. The joint posterior distribution of the two parameters conditional on the data were estimated via incremental mixture importance sampling (IMIS) [38]. The joint distribution was found to be unimodal and strongly peaked; therefore, parameters for subsequent simulations were set at the maximum a posteriori estimate (joint posterior distribution, Additional file 1: Figure S2A; marginal distributions, Figure S2B, S2C in Additional file 1). For consistency, the values for these parameters were also used in the spreadsheet model. The posterior estimate of the reduction in susceptibility to reinfection was similar to previous estimates of bacille Calmette-Guerin (BCG) protection against active disease, 0.58 (95% CI 0.35–1.01) [39]. Technical details regarding the calibration procedure are available in Additional file 1.

To measure the incidence attributable to mine workers using the individual-based model, we simulated two counterfactual scenarios: first, having no *Mtb* transmission in the mines and, second, having no *Mtb* transmission in any area. These scenarios were identical to the baseline scenario until simulated year 2012 when *Mtb* transmission was stopped in the model. Other processes such as disease progression continued unchanged. The numbers of new cases of active disease between simulated years 2014 and 2019 were counted for each counterfactual scenario and compared to the baseline scenario. This calculation was repeated for each residency group in the model.

## Results

### Most force of infection in communities is attributable to local residents

We used a static risk model to calculate the FOI and number of transmission events (new infections) in different mining-related communities in South Africa and predict the near-term (following-year) incidence in these communities (Fig. 1a). The model accounted for a number of factors including a higher rate of *Mtb* transmission due to environmental factors and the amount of time that residents reported spending in their own versus other communities, where mixing was assumed to be proportional to the time spent in each community (Fig. 1b).

As a check on the static risk model, we compared FOI output from the model to data on the annual risk of TB infection (ARTI) in children. Under baseline parameters, we estimated FOI to be 21.2% (95% CI 16.4–26.1%) in mine workers, 4.3% (95% CI 4.3–4.3%, indicating a difference of < 0.05%) in peri-mining residents, 5.8% (95% CI 5.8–5.8%) in labor-sending residents, and 3.5% (95% CI 3.5–3.5%) in other South African residents (Table 1, Additional file 1: Table S5). CIs were derived by sampling parameter values for mine-specific transmission and immunity following previous infection. The FOI estimate for other South African residents in the model was found to be consistent with available ARTI measurements: 2.5–4.2% (across Western Cape, 2005, [40]), 3.8–4.5% (in Cape Town, 2005, [41]), 3.9–4.8% (in Cape Town, 2009, [42]), and 2.1–5.2% (in Johannesburg, 2013, [43]).

**Table 1:** Force of infection (per-susceptible rate of infection) attributable to each population

	From mining residents	From peri-mining residents	From labor-sending residents	From other SA residents	From all residents
Among mining residents	2.00 × 10^−1^ (94.0%)	4.41 × 10^−3^ (2.1%)	6.98 × 10^−3^ (3.3%)	1.32 × 10^−3^ (0.6%)	2.12 × 10^−1^ (100%)
Among peri-mining residents	2.48 × 10^−3^ (5.8%)	3.90 × 10^−2^ (91.5%)	5.73 × 10^−4^ (1.3%)	5.93 × 10^−4^ (1.4%)	4.27 × 10^−2^ (100%)
Among labor-sending residents	2.09 × 10^−3^ (3.6%)	3.04 × 10^−4^ (0.5%)	5.52 × 10^−2^ (94.7%)	6.88 × 10^−4^ (1.2%)	5.83 × 10^−2^ (100%)
Among other SA residents	3.88 × 10^−5^ (0.1%)	3.15 × 10^−4^ (0.2%)	6.80 × 10^−5^ (0.2%)	3.45 × 10^−2^ (98.8%)	3.51 × 10^−2^ (100%)

Per-annum rate and percentage of total from all groups using mean of Monte Carlo simulations from the spreadsheet model

Using the static risk model, we then calculated the fraction of FOI in each community attributable to each residency group. We estimated that the majority of each community’s FOI was attributable to local residents: 93.9% (95% CI 92.4–95.1%), 91.5% (95% CI 91.4–91.5%), 94.7% (95% CI 94.6–94.7%), and 98.8% (95% CI 98.8–98.8%) in mining, peri-mining, labor-sending, and other SA communities, respectively (Table 1, Additional file 1: Table S5). Despite the amount of time mine workers were assumed to spend in other areas (up to 20% per annum), the FOI in peri-mining, labor-sending, and other SA communities attributable to mine workers was estimated to be 5.8% (95% CI 5.8–5.8%), 3.6% (95% CI 3.5–3.6%), and 0.1% (95% CI 0.1–0.1%), respectively.

### Gold mine workers contribute more TB infections per capita than other residents

Using the preceding FOI and data on susceptible individuals, i.e., either uninfected or latently infected but susceptible to reinfection, we estimated the number of new infections expected to occur in each community and compared these results to published TB incidence for different communities. For mine workers and the overall population, we estimated TB incidence to be 2963 (95% CI 2208–3858) and 989 (95% CI 980–1000) per 100,000 individuals, respectively. These were similar to published values for these communities, 2957 (in control cluster mines during the Thibela TB study, between 2006 and 2010 [21]) and 977 (717–1276) (in South Africa, 2008 [20, 44]) per 100,000 (Additional file 1: Table S6).

Using the static risk model, we also estimated the fraction of infections occurring each year attributable to each residency group. Out of the overall number of new infections occurring in South Africa per annum, we estimated that 4.0% (95% CI 2.6–5.8%), 5.0% (95% CI 4.5–5.5%), and 9.0% (95% CI 8.8–9.1%) were attributable to mining, peri-mining, and labor-sending residents, respectively (Table 2, Additional file 1: Table S7). When scaled to the fraction of the overall population in South Africa that each group represents, mine workers, peri-mining residents, and labor-sending residents contributed 4.32 (95% CI 2.77–6.15), 1.21 (95% CI 1.09–1.34), and 1.39 (95% CI 1.36–1.41) times as many infections as South Africans as a whole (Table 2, Additional file 1: Table S7). Similarly, when scaled to the fraction of the overall number of prevalent cases in South Africa found in each group, mine workers, peri-mining residents, and labor-sending residents contributed 1.62 (95% CI 1.04–2.30), 1.14 (95% CI 1.02–1.25), and 1.07 (95% CI 1.05–1.09) times as many infections as South Africans as a whole (Table 2, Additional file 1: Table S7). Therefore, while mine workers contribute a larger number of infections on a per-capita or per-prevalent case basis than other South Africans, the majority of these infections occur among mine workers themselves.

**Table 2:** New infections in all South Africa attributable to each population

	From mining residents	From peri-mining residents	From labor-sending residents	From other SA residents	From all residents
New infections among all SA residents	6.25 × 10^4^ (4.1%)	7.77 × 10^4^ (5.0%)	1.38 × 10^5^ (9.0%)	1.26 × 10^6^ (81.9%)	1.54 × 10^6^ (100%)
Population size of attributable source	4.85 × 10^5^ (0.9%)	2.14 × 10^6^ (4.1%)	3.35 × 10^6^ (6.5%)	4.58 × 10^7^ (88.4%)	5.18 × 10^6^ (100%)
Ratio of new infection %:population %	4.33	1.22	1.39	0.93	1.00
Prevalence est. in attributable source	1.04 × 10^4^ (2.5%)	1.84 × 10^4^ (4.4%)	3.47 × 10^4^ (8.4%)	3.52 × 10^5^ (84.7%)	4.15 × 10^5^ (100%)
Ratio of new infection %:prevalence %	1.62	1.14	1.07	0.97	1.00

Number of cases and percentage of total; ratio of percentage of total infections to percentage of total population that each group represents; and ratio of percentage of total infections to percentage of total prevalence that each group represents, using mean of Monte Carlo simulations from the static risk model

### Local recent transmission is the source of the majority of incident TB cases in gold mines

**Fig. 2:**
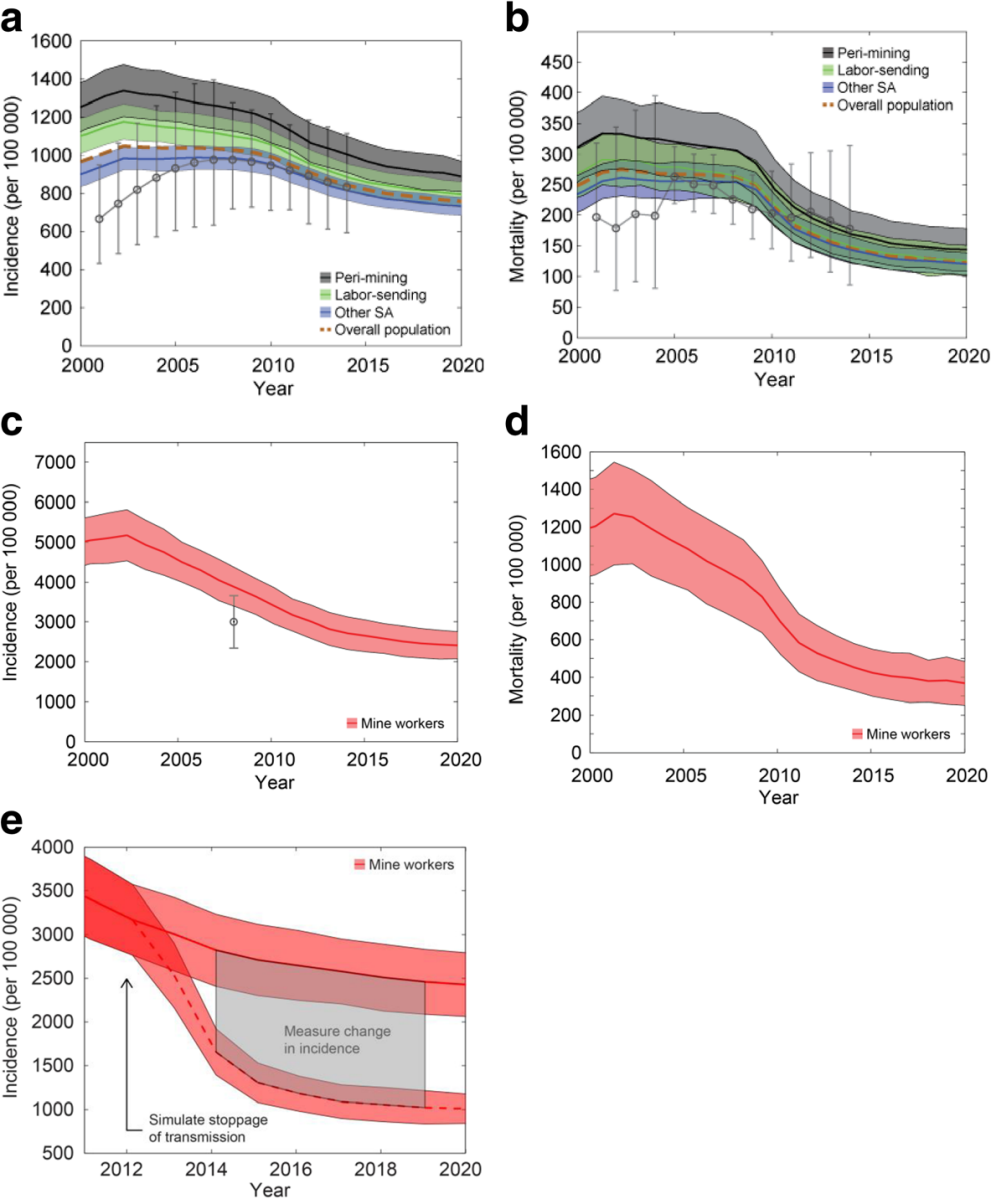
Simulated time series of the TB epidemic in different communities in South Africa. Means and 95% CIs were derived from 200 stochastic realizations of the model where input parameters were set at the mode of the posterior distribution of two calibration parameters. **a** TB incidence in peri-mining, labor-sending, and other South Africa residents. **b** TB mortality in peri-mining, labor-sending, and other South Africa residents. In **a** and **b**, the population-weighted mean of the four populations in the model is also shown. **c** TB incidence in mine workers. **d** TB mortality in mine workers. **e** Methodology for computing the fraction of incidence attributable to recent *Mtb* transmission in the mines. The *upper curve* is identical to the curve in **c**, while the *lower curve* represents the mean and 95% CI of stochastic realizations that were identical to **c** until simulated year 2012, after which *Mtb* transmission from mine workers was stopped but all other aspects of the model remained unchanged. Attribution was calculated from the difference in incidence between simulated years 2014 and 2019

To measure the impact of *Mtb* transmission in the mines on incidence, we used a dynamic, individual-based model of TB in South Africa. In this model we accounted for longer-term demographic changes and additional pathways leading to active disease including reactivation from transmission occurring over a longer time window. We calibrated the model to several epidemiological indicators including incidence and mortality over multiple years [21, 37]. Model estimates of TB incidence and mortality overlapped published ranges, both at the country level (Fig. 2a, b; Additional file 1: Figure S3A, S3B) and at the mining level (Fig. 2c, d; Additional file 1: Figure S3C, S3D). In particular, model incidence reproduced measurements from the Thibela TB study, showing a threefold higher incidence in the mines compared to South Africa overall (Fig. 2a, c). Model incidence in the mines preceding the Thibela TB study was exceeded 4000 per 100,000 (Fig. 2c), consistent with previous studies on mine workers [45]. Model mortality due to TB among mine workers was approximately 1% per annum (Fig. 2d), which was consistent with a range that includes the 0.9% all-cause mortality rate and 4.3% all-cause mortality-plus-medically boarded rate observed as a secondary outcome of the Thibela TB study [21]. As an additional test of the model, including South Africa-specific parameters derived from calibration, we used the model to simulate the Thibela TB study intervention of widely available preventive therapy. Following a cessation of the intervention, we observed a rebound in model incidence similar to the rebound observed during the Thibela TB study (Additional file 1: Figure S4A).

To estimate the fraction of incident cases attributable to gold mines, we simulated a counterfactual scenario of stoppage of *Mtb* transmission in mining areas and measured the subsequent change in incidence over several years (Fig. 2e). By doing so, we estimated that recent *Mtb* transmission in the mines contributed 58.2% (95% CI 57.8–58.9%), 4.8% (95% CI 4.3–5.2%), and 4.9% (4.4–5.2%) of the TB incidence in mining, peri-mining, and labor-sending residents, respectively (Table 3). Among other residents of South Africa, the counterfactual scenario had a smaller effect that resulted in a time course that overlapped the baseline scenario at all time points, i.e., within the stochastic noise of the simulation (Table 3). In South Africa as a whole, the fraction of TB incidence due to recent *Mtb* transmission in the mines was estimated to be 2.4% (95% CI 1.4–3.3%) (Table 3). To test the robustness of this measurement, we performed a series of one-way sensitivity analyses based on varying the infectiousness in each community separately (mining, peri-mining, labor-sending, and other South Africa). In all cases, the resulting fraction of TB incidence due to recent *Mtb* transmission in the mines was found to vary maximally between 1 and 4% (Additional file 1: Figure S5).

**Table 3:** Incidence attributable to recent transmission in mining areas

	From recent transmission in mining areas (col. 1)	From recent transmission in all areas (col. 2)	Fraction of all incidence due to recent transmission that is attributable to mining areas (col. 1/col. 2)
Incidence among mining residents	58.2% (57.8%, 58.9%)	63.0% (58.5%, 67.7%)	92.5% (92.1%, 92.9%)
Incidence among peri-mining residents	4.8% (4.3%, 5.2%)	39.3% (35.4%, 43.2%)	11.4% (10.2%, 12.7%)
Incidence among labor-sending residents	4.9% (4.4%, 5.2%)	39.5% (35.9%, 43.2%)	11.6% (10.7%, 12.6%)
Incidence among other SA residents	0.0% (−0.2%, 0.3%)	36.3% (33.7%, 38.9%)	−0.4% (−1.3%, 0.4%)
Incidence among all residents	2.4% (1.4%, 3.3%)	37.4% (35.2%, 40.0%)	3.7% (3.0%, 4.4%)

Using the individual-based model, transmission ceased in mines (column 1) or in the entire population (column 2) beginning in year 2012, and differences in incidence were measured between years 2014 and 2019. Mean and 95% CI are shown using parameters with highest likelihood from model calibration

As a second counterfactual scenario, we simulated stoppage of transmission in all areas of South Africa and calculated the fraction of incident cases attributable to recent transmission from any source. Among mining residents, 63.0% (95% CI 58.5–67.7%) of the incident cases were predicted to result from recent transmission, 92.5% (95% CI 92.1–92.9%) of which were attributable to recent transmission in mining areas (Table 3). In contrast, among all South African residents, 37.4% (95% CI 35.2–40.0%) of the incident cases were predicted to result from recent transmission, 3.7% (95% CI 3.0–4.4%) of which were attributable to recent transmission in mining areas (Table 3). These figures were consistent with local *Mtb* transmission in mines being the source for the majority of incident TB cases in the mines but only a small fraction of the incident TB cases in the remainder of the country.

## Discussion

Using two different modeling approaches, we found that gold mine workers are likely to be contributing to the TB burden in South Africa but primarily at the level of their own communities and not the larger population of South Africa, owing to the generalized nature of the TB epidemic in South Africa. Using a static risk model, we captured several parameters that determine the extent of *Mtb* transmission from mine workers: the size of different populations with whom mine workers interact, the prevalence of latent infection and active disease in each population, and the amount of time that residents from different populations spend with each other. Our model suggests that gold mine workers who number less than 0.5 M (< 1% of the population in South Africa) contribute approximately 4% of new infections in South Africa per annum. By comparison, residents in peri-mining and labor-sending areas who number approximately 2.1 M and 3.4 M (4% and 7% of the population) contribute approximately 5% and 9% of new infections in South Africa per annum, respectively. Therefore, mine workers contribute a disproportionately large number of new infections, as one might expect given their higher rates of disease and the setting in which they work. However, given their mixing patterns and other factors which we included in the model, we found that the effect is mostly at the level of their own communities. These factors include the amount of time that residents spend in other communities, which we estimated to be less than 25% per year, and the limited number of susceptibles available in other communities. For example, in high-burden areas such as peri-mining and labor-sending areas, more than 50% of the population may already be latently infected, reflecting a high FOI in these areas [42, 46].

We obtained similar results with an individual-based model which we used to simulate a counterfactual scenario of curtailed *Mtb* transmission in the mines for a period of more than 2 years. Using this approach, we estimated that 4% of the incidence in all of South Africa could be traced to recent transmission in the mines, similar to the attributable fraction of new infections. However, among mine workers themselves, greater than 50% of the incident cases could be traced to recent transmission in the mines, suggesting that ongoing transmission among mine workers continues to have a significant effect. This was consistent with results from Godfrey-Faussett and colleagues, who genotyped *Mtb* strains from mine workers and found that at least 50% of TB cases were due to transmission within the mines [47], as well as other studies showing a high degree of strain clustering in different parts of South Africa [46, 48, 49]. However, a more recent study of the Thibela TB study site by Mathema and colleagues has suggested that the fraction of incident TB cases in the mines due to recent infection may be lower than previously measured [50]. Additional work is needed to explain the differences in these results and connect results such as ours, based on simulation and counterfactuals, to results based on genetic clustering and molecular epidemiology.

Nonetheless, our results suggest that curtailing transmission in the mines may have a measurable impact on the number of new cases of TB disease in the mines on a relatively short time frame, within 5 years or less. This is similar to the time frame posited by Vynnycky and colleagues, who used modeling to simulate a set of mine-targeted interventions such as reduced treatment delay and scaled-up ART and found it was possible to obtain a significant impact [15]. Given our results and those of Vynnycky et al., health officials may wish to consider measuring the extent of recent transmission, such as through *Mtb* strain genotyping, on an ongoing basis. A decrease in the proportion of cases that cluster genotypically is expected to accompany effective programs and would provide additional evidence of the effectiveness of TB control programs.

Our study complements past efforts to measure the association between TB burden and mining such as the study by Stuckler and colleagues [51]. In that study, each 10% increase in mining production was associated with a 0.9% increase in TB incidence [51]. Our approach did not include mining production as a covariate, precluding a direct comparison of the results. In addition, we focused on the ongoing contribution of mine conditions, which differed from the focus of Stuckler et al. on historical mining production. Despite these differences, both our study and that of Stuckler et al. point to the need to consider mining in a larger context, whether that be population mixing or other comorbidities. For example, Stuckler et al. found that most of the effect of mining on TB was mediated by HIV prevalence; controlling for HIV greatly reduced the association with mining [51]. In our models, HIV plays a similarly large role and increases the activation rate of latent disease in all groups including mine workers. The large effect of HIV relative to mining production can also be seen directly by comparing the time courses for mining production, HIV prevalence, and TB incidence in South Africa. While mining production has decreased over the last two decades [52], TB incidence has more closely mirrored HIV prevalence, only beginning to decline after 2010 [53]. As ART usage continues to increase and HIV prevalence stabilizes, it will be interesting to observe whether decreases in mining production have a more discernible effect on TB incidence. Additional questions include whether decreasing mining production or *Mtb* transmission in the mines would have a different effect depending on HIV prevalence 5, 10, or 15 years in the future and more generally whether the impact of hotspot-targeted approaches depends on prior HIV control and whether hotspot targeting should be coordinated with HIV control programs. We plan to explore these questions in future applications of the model.

Our study also contributes to the growing literature on using quantitative approaches to investigate potential TB hotspots. Recently, Dowdy and colleagues used a model to study high TB burden areas in Rio de Janeiro, Brazil [54]. In that study, areas that comprised 6% of the city population were found to contribute 35% of the new infections in the city, resulting in a 5.8:1 attributable infection:population size ratio. This compares to the 4.3:1 attributable infection:population size ratio that we found for mine workers (Table 2). As the quality of TB monitoring and evaluation improves globally, it may be useful to define a set of functional criteria for TB hotspots, e.g., what attributable infection:population size ratios qualify an area to be a hotspot and how many susceptibles need to reside in the larger population for a hotspot to pose a risk. To encourage discussions in this area, we have made many of these outputs, along with modifiable assumptions, accessible in our spreadsheet model.

While we accounted for several factors in our study, including HIV, silicosis, and population mixing, a number of assumptions would benefit from additional study. For example, our results assume that the prevalence of latent infection and active disease in the peri-mining and labor-sending areas was at least as high as those found in the general population of South Africa. While this is supported by historical data [1, 2] and available case notification data [55], our estimates could be improved with accurate measurements in these areas, such as may become available from future prevalence surveys in South Africa. The number of populations that are included in the models could also be expanded to include foreign workers. Although the proportion of workers from countries outside of South Africa has decreased in recent decades, a more comprehensive accounting should include other countries in southern Africa including Lesotho, Swaziland, and Mozambique [56]. Finally, how we represent mixing could also be refined to account for different scales. While we informed mixing in our models using tourism and labor migration data, these provide only a proxy of mixing and exclude more local influences, such as interactions within mining areas and hostels and on public transport [57, 58]. Investigating *Mtb* transmission at more granular levels may lead to more actionable findings for mitigating risk.

Despite these caveats, we believe our models account for the main factors likely to govern the contribution of gold mines to the TB epidemic: the size of the mine worker population, the TB burden in mine workers and other groups, and the amount of time mine workers spend in different areas. Together these factors suggest *Mtb* transmission in gold mines continues to feed infections in mines and mining-related communities but to a much smaller extent in the country as a whole. In evaluating the impact of interventions designed to curtail transmission in the mines, the effect on both scales should be considered.

## Conclusions

Using two models that integrate diverse types of data, we estimate that gold mine workers contribute a disproportionately large number of *Mtb* infections in South Africa on a per-capita basis. However, due to their relatively small population and the generalized nature of the TB epidemic in South Africa, gold mine workers contribute only a small fraction of the total number of *Mtb* infections in South Africa. Our results suggest efforts at curtailing transmission in the mines may have limited impact at the country level despite a potentially significant impact on a relatively short time frame in the mines themselves.

## Additional file

Additional file 1: Supplemental materials, figures, and tables. (DOCX 2293 kb)

## Abbreviations

ART: Antiretroviral therapy
95% CI: 95% confidence interval
DOTS: Directly observed therapy short-course
FOI: Force of infection
LTBI: Latent tuberculosis infection
M: Million
*Mtb*: *Mycobacterium tuberculosis*
PAF: Population attributable fraction
TB: Tuberculosis
WAIFW: Who acquires infection from whom
