# Correction to: Small contribution of gold mines to the ongoing tuberculosis epidemic in South Africa: a modeling-based study

**DOI:** 10.1186/s12916-018-1238-9

**Published:** 2018-12-28

**Authors:** Stewart T. Chang, Violet N. Chihota, Katherine L. Fielding, Alison D. Grant, Rein M. Houben, Richard G. White, Gavin J. Churchyard, Philip A. Eckhoff, Bradley G. Wagner

**Affiliations:** 1 Institute for Disease Modeling, Bellevue, Washington USA; 2 Aurum Institute, Johannesburg, South Africa; 3 School of Public Health, Faculty of Health Sciences, University of Witwatersrand, Johannesburg, South Africa; 4 Foundation for Innovative New Diagnostics, Geneva, Switzerland; 5 Department of Infectious Disease Epidemiology, London School of Hygiene and Tropical Medicine, London, UK; 6 Department of Clinical Research, London School of Hygiene and Tropical Medicine, London, UK; 7 Africa Health Research Institute, School of Nursing and Public Health, University of KwaZulu-Natal, Durban, South Africa; 8 TB Modelling Group, CMMID, TB Centre, London School of Hygiene and Tropical Medicine, London, UK; 9 Advancing Treatment and Care for TB/HIV, South African Medical Research Council, Johannesburg, South Africa; 10 Institute for Disease Modeling, Bellevue, Washington USA

**Correction to: BMC Med**

**https://doi.org/10.1186/s12916-018-1037-3**

The original article [1] did not contain comprehensive information regarding two authors’ affiliations that may be considered a potential competing interest. VN Chihota and GJ Churchyard are affiliated with the Aurum Institute. The Aurum Institute was founded in 1998 as a subsidiary of Anglogold Ashanti Health Services to meet the needs of AngloGold Ashanti with respect to TB, HIV, and occupational lung diseases. The name was changed to Aurum Institute in 2005 when it became independent from AngloGold Ashanti Health Services. The Aurum Institute is now a registered not-for-profit, public benefit organisation that receives no funding from the mining industry.
