# Supplementary material for: Small contribution of gold mines to the ongoing tuberculosis epidemic in South Africa: a modeling-based study
Source: BMC Med. 2018 Apr 12;16:52. doi: 10.1186/s12916-018-1037-3 (PMC5896106; doi:10.1186/s12916-018-1037-3)
Supplement: Supplementary file 1 — Supplemental materials, figures, and tables. (DOCX 2293 kb) [file 12916_2018_1037_MOESM1_ESM.docx]

# **Supplement to "Small contribution of gold mines to the ongoing tuberculosis epidemic in South Africa: A modeling-based study"**

# Methods

## Static risk model availability

## The spreadsheet model is available as a Microsoft Excel file in a GitHub repository (https://github.com/SCTX/mining_contribution).

## Individual-based model availability

The model was written in C++ and is based on the TB model in the EMOD modeling framework which was described previously [[1,2]](https://paperpile.com/c/5kxPgO/Kgtgz+NkfKo). Files are available in a GitHub repository (https://github.com/SCTX/mining_contribution). Model parameters were specified in three json-formatted input files available with the source code: a demographics file which specifies population seeding and attributes assigned to individuals in the model; a configuration file which specifies rate constants and other parameters for TB natural history, risk factors, and anti-TB drug effects; and a campaign file which specifies the timing and extent of TB and HIV outbreaks in the model and interventions such as DOTS and ART.

### Individual-based model parameters

### Population demographics

Individuals were born at each time step with a probability that corresponded to the population growth rate varying between 2.2% and 3.1% annually. At birth individuals were assigned to one of four residency groups (mining, labor-sending, peri-mining, or remaining South Africa) with probability 0%, 16.7%, 33.3%, and 50%, respectively. Individuals were also assigned to high- or low-quality health care access with probability 65% and 35%, respectively [[3]](https://paperpile.com/c/5kxPgO/DO3Xq). Individuals died from non-TB causes in the model with a probability that varied by age from approximately 1.5% annually between 0 and 5 years to 85% annually between 100 and 105 years with no individual allowed to live longer than 105 years.

The model-predicted age pyramid for 2016 was compared to current estimates and showed good agreement with population demographics (Figure S1A). The age distribution of mine workers in the model differed from that of other populations in the model due to migration to and from labor-sending communities (Figure S1B). The age distribution of mine workers in the model reflected the age distribution of the mining workforce measured in the Thibela TB study where the majority of individuals were aged between 20 and 49 [[4]](https://paperpile.com/c/5kxPgO/FVzWj).

### TB natural history

Disease progression in the model was parameterized through rate constants representing the mean durations between disease states: susceptible, latently infected, pre-symptomatic active, symptomatic active, and recovered (Table S1). We assumed that the processes corresponding to transitions between these states occur similarly across human populations and therefore based parameter values on a previous version of the model calibrated to TB in China [[1]](https://paperpile.com/c/5kxPgO/Kgtgz) except in the case of base infectivity whose value was derived through calibration.

| ***Parameter*** | ***Definition*** | ***Baseline value*** |
| --- | --- | --- |
| TB_Fast_Progressor_Fraction_Child | Fraction of under-age 15 who are designated “fast progressors” | 5% |
| TB_Fast_Progressor_Fraction_Adult | Fraction of over-age 15 who are designated “fast progressors” | 11% |
| TB_Fast_Progressor_Rate | Rate from latent TB to active presymptomatic TB for “fast progressors” | 2.0 yr^-1^ |
| TB_Slow_Progressor_Rate | Rate from latent TB to active presymptomatic TB for “slow progressors” | 0.005 yr^-1^ |
| TB_Latent_Cure_Rate | Rate from latent TB to uninfected by spontaneous cure | 0 yr^-1^ |
| TB_Base_Infectivity_Presymptomatic | R0 for active presymptomatic TB | 7.0 x 10^-3^ d^-1^ |
| TB_Presymptomatic_Rate | Rate from active presymptomatic TB to fully active TB | 2.7 x 10^-3^ d^-1^ |
| TB_Presymptomatic_Cure_Rate | Rate from active presymptomatic TB to uninfected by spontaneous cure | 0 d^-1^ |
| TB_Base_Infectivity | R0 for fully active TB | 2.2 x 10^-2^ d^-1^ |
| TB_Active_Mortality_Rate | Rate of mortality with active TB | 4.1 x 10^-4^ d^-1^ |
| TB_Active_Cure_Rate | Rate from active TB to uninfected by spontaneous cure | 2.7 x 10^-4^ d^-1^ |
| TB_Smear_Positive_Fraction_Child | Fraction of under-age 15 who are designated smear-positive | 2.5 x 10^-1^ |
| TB_Smear_Positive_Fraction_Adult | Fraction of over-age 15 who are designated smear-positive | 6.5 x 10^-1^ |
| TB_Smear_Negative_Infectious_Multiplier | Multiplier for TB_Base_Infectivity for smear-negative | 1.5 x 10^-1^ |
| TB_Smear_Negative_Mortality_Multiplier | Multiplier for TB_Active_Mortality_Rate for smear-negative | 1.5 x 10^-1^ |
| TB_Extrapulmonary_Fraction_Child | Fraction of under-age 15 who are designated extrapulmonary | 4.0 x 10^-1^ |
| TB_Extrapulmonary_Fraction_Adult | Fraction of over-age 15 who are designated extrapulmonary | 1.0 x 10^-1^ |
| TB_Extrapulmonary_Mortality_Multiplier | Multiplier for TB_Active_Mortality_Rate for extrapulmonary | 1.5 x 10^-1^ |

Table S1. TB natural history parameters in the individual-based model

New parameters for the EMOD TB model represented factors relevant to South Africa, namely HIV and silicosis. These parameters modify the rate of infection, disease progression, and mortality (Table S2). We assumed that processes underlying these parameters occur similarly across populations and therefore base their values on systematic reviews.

| ***Parameter*** | ***Definition*** | ***Baseline value*** |
| --- | --- | --- |
| CD4_Strata_Activation | CD4 levels below which a modified slow progressor rate is used | {3.0 x 10^-2^,  3.5 x 10^2^,  5.0 x 10^2^,  2.0 x 10^5^} |
| TB_CD4_Activation_Vector | Slow progressor rate (i.e., transition rate from latent TB to active TB) for different CD4 levels | {5.5 x 10^-4^ d^-1^,  3.0 x 10^-4^ d^-1^,  1.0 x 10^-4^ d^-1^,  1.4 x 10^-5^ d^-1^} |
| TB_CD4_Infectiousness | Multiplier on R0 for active TB in HIV-TB co-infected | 6.0 x 10^-1^ |
| Coinfection_Mortality_Rate_Off_ART | Rate of mortality of HIV-TB co-infected while not on ART | 1.6 x 10^-4^ d^-1^ |
| Coinfection_Mortality_Rate_On_ART | Rate of mortality of HIV-TB co-infected while on ART | 8.0 x 10^-5^ d^-1^ |
| Silicosis_Rate | Rate of developing silicosis | 2.5 x 10^-5^ d^-1^ |
| Silicosis_TB_Rate | Rate from latent TB to active TB for silicotics | 2.0 x 10^-5^ d^-1^ |
| Silicosis_TB_HIV_Risk | Risk of progression from latent TB to active TB among HIV-positives (as a multiple of the rate in non-silicotic HIV-positive individuals) | 3 |

Table S2. Additional parameters for South Africa-relevant risk factors in the individual-based model

### Simulation of epidemiological events

### Several discrete epidemiological events were represented in the model. After an initial burn-in period of 100 model years, steady state behaviors were obtained for incidence, prevalence, and mortality. At this point, HIV was seeded into the model, corresponding to simulation year 1985. DOTS was made available in simulation year 2002 and ART in simulation year 2007.

### HIV infection

### Individuals in the model were assigned HIV infection with probabilities that varied as a function of age, gender, and date. These probabilities were set at 0% during model burn-in then made available starting from simulation year 1985. HIV incidence was specified as an input to the model based on the South Africa EMOD HIV model with prevalence determined by ART coverage, TB transmission dynamics, and TB treatment. Individuals with HIV in the model were assigned a CD4 count that decreased linearly with time in the absence of ART. When ART was given to an individual in the model, it effected an increase in CD4 count as specified by the EMOD HIV model [[5]](https://paperpile.com/c/5kxPgO/kLXAT).

Country-level HIV prevalence for 15-49 year-olds and overall ART coverage are shown in Figure S1C and S1D, respectively. We assumed that HIV prevalence among mine workers was similar to the age-stratified HIV prevalence in the overall population and that ART scale-up in mining areas also followed trends at the country-level. HIV prevalence for 2012 in the model (mean: 17.0%, CI95: 16.4-17.7%) was consistent with published estimates for that year (mean: 18.8%, CI95: 17.5-20.3%) [[6]](https://paperpile.com/c/5kxPgO/giM0q).

### TB treatment

Individuals in the model with active TB were eligible to receive treatment. Prior to the availability of DOTS, we assumed that only partially effective treatment was available. After the introduction of DOTS, 65% and 35% of individuals were given access to high- and low-quality TB treatment, respectively. Individuals who developed active symptomatic TB while having access to high- or low-quality TB treatment were assumed to access care after a mean duration of 4 months [[7]](https://paperpile.com/c/5kxPgO/Tj15C) or 12 months [[8]](https://paperpile.com/c/5kxPgO/u82i9), respectively. Upon accessing care, individuals received a GeneXpert-like test with 98% and 79% sensitivity for smear-positive and smear-negative disease, respectively [[9]](https://paperpile.com/c/5kxPgO/5v3KX). With a positive result, individuals were assumed to begin treatment which had the effect of returning individuals to a cleared, uninfected state identical to the susceptible state with partial immunity to reinfection or a latently infected state, according to reported treatment success rates (Table S3). We assumed the processes underlying these parameters were specific to South Africa and therefore derived values from South Africa-specific sources, except in the case of immunity. Partial immunity was assumed to reduce the rate of subsequent infection where the magnitude of the reduction was set by calibration to population-level epidemiological data.

| ***Parameter*** | ***Definition*** | ***Baseline value*** |
| --- | --- | --- |
| TB_Drug_Clearance_Rate_Pre-DOTS_ | Rate from active TB to clearance in pre-DOTS era | 5.9 x 10^-3^ d^-1^ |
| TB_Drug_Inactivation_Rate_Pre-DOTS_ | Rate from active TB to latent TB in pre-DOTS era | 6.1 x 10^-4^ d^-1^ |
| TB_Drug_Clearance_Rate_DOTS-LQ_ | Rate from active TB to clearance in DOTS era with low-quality treatment | 5.9 x 10^-3^ d^-1^ |
| TB_Drug_Inactivation_Rate_DOTS-LQ_ | Rate from active TB to latent TB in DOTS era with low-quality treatment | 6.1 x 10^-4^ d^-1^ |
| TB_Drug_Clearance_Rate_DOTS-HQ_ | Rate from active TB to clearance in DOTS era with high-quality treatment | 7.9 x 10^-3^ d^-1^ |
| TB_Drug_Inactivation_Rate_DOTS-HQ_ | Rate from active TB to latent TB in DOTS era with high-quality treatment | 6.7 x 10^-4^ d^-1^ |

Table S3. Treatment-related parameters in the individual-based model

Input values were equivalent in the model to a treatment cure and success proportions of 58% and 69%, respectively, in the pre-DOTS era. These proportions were assumed to be unchanged in the DOTS era with low-quality treatment. However, these proportions increased to 71% and 77%, respectively, in the DOTS era with high-quality treatment, similar to observed rates for South Africa [[10]](https://paperpile.com/c/5kxPgO/VwUtH).

### Short-term mixing between groups

The rate at which individuals were infected in the model and moved from susceptible to latently infected status depended on their residency status. The model accounts for the probability with which an individual of a residency group *i* may encounter an individual of a residency group *j* in a community *k* (mining, peri-mining, labor-sending, or other South African community). For example, a susceptible peri-mining resident can be infected by a mine worker with active TB when the former visits a mining area, the latter visit a peri-mining area, or both visit an area that is neither mining nor peri-mining. These relationships are represented by a who-acquires-infection-from-whom (WAIFW) matrix where each element is defined by

[
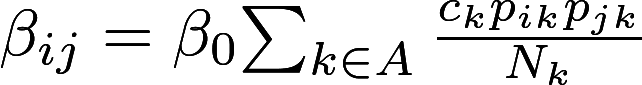
](http://api.gmath.guru/cgi-bin/gmath?%5Cdpi%7B480%7D%5Cbeta_%7Bij%7D%3D%5Cbeta_%7B0%7D%24%5Csum_%7Bk%20%5Cin%20A%7D%20%5Cfrac%7Bc_%7Bk%7Dp_%7Bik%7Dp_%7Bjk%7D%7D%7BN_%7Bk%7D%7D)

This follows a frequency-based formulation where each element 𝛽*_ij_* represents the per-year probability that a susceptible resident of row *i* will be infected by an infective resident of column *j*. 𝛽*_0_* is the base transmission rate, or R0 per year; *k* is one of the communities from the set of all communities *A*; c*_k_* is a multiplier on transmission specific to the community *k*; *p_ik_* is the fraction of time that individuals from residency group *i* spend in community *k*; and *p_jk_* is the fraction of time that individuals from residency group *j* spend in community *k* (Table S4). These values on social mixing were specific to South Africa and therefore derived from South Africa-specific sources such as tourism surveys.

*N_k_* is the effective population size present in area *k*, summing the number of residents present in their own community *k* and the number of visitors to a community *k* at any given time, such that:

[
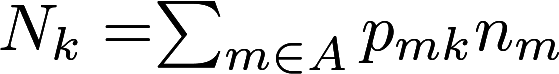
](http://api.gmath.guru/cgi-bin/gmath?%5Cdpi%7B480%7DN_%7Bk%7D%3D%24%5Csum_%7Bm%20%5Cin%20A%7D%7Bp_%7Bmk%7Dn_%7Bm%7D%7D)

where *m* is one of the communities from the set of all communities *A*, *p_mk_* is the fraction of time that a resident of community *m* spends in community *k* and *n*_m_ is the number of residents counted by census or trade industry. Alternatively, *N_k_* can be viewed as the size of community *k* over the course of one year in person-years.

Finally the force of infection (per-susceptible rate of infection) experienced by a residency group *i* attributable to a residency group *j* is given by

[
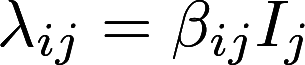
](http://api.gmath.guru/cgi-bin/gmath?%5Cdpi%7B480%7D%5Clambda_%7Bij%7D%3D%5Cbeta_%7Bij%7D%20I_%7Bj%7D)

where *I_j_* represents the number of prevalent TB cases in residents of community *j*.

| ***Parameter*** | ***Definition*** | ***Baseline value*** |
| --- | --- | --- |
| *p*_M-PM_ | Fraction of time that mine workers spend in peri-mining communities | 1.1 x 10^-1^ |
| *p*_M-LS_ | Fraction of time that mine workers spend in labor-sending communities | 1.2 x 10^-1^ |
| *p*_M-SA_ | Fraction of time that mine workers spend in the remainder of South Africa | 1.0 x 10^-2^ |
| *p*_PM-M_ | Fraction of time that peri-mining residents spend in mining communities | 1.2 x 10^-4^ |
| *p*_PM-LS_ | Fraction of time that peri-mining residents spend in labor-sending communities | 2.4 x 10^-3^ |
| *p*_PM-SA_ | Fraction of time that peri-mining residents spend in the remainder of South Africa | 1.0 x 10^-2^ |
| *p*_LS-M_ | Fraction of time that labor-sending residents spend in mining communities | 3.0 x 10^-4^ |
| *p*_LS-PM_ | Fraction of time that labor-sending residents spend in peri-mining communities | 5.9 x 10^-3^ |
| *p*_LS-SA_ | Fraction of time that labor-sending residents spend in the remainder of South Africa | 1.0 x 10^-3^ |
| *p*_SA-M_ | Fraction of time that other residents of South Africa spend in mining communities | 3.0 x 10^-5^ |
| *p*_SA-PM_ | Fraction of time that other residents of South Africa spend in peri-mining communities | 7.5 x 10^-3^ |
| *p*_SA-LS_ | Fraction of time that other residents of South Africa spend in labor-sending communities | 1.1 x 10^-3^ |
| c_M_ | Multiplier on infectiousness for active TB in mining communities | 2.7 x 10^0^ |
| c_PM_ | Multiplier on infectiousness for active TB in peri-mining communities | 1.2 x 10^0^ |
| c_LS_ | Multiplier on infectiousness for active TB in labor-sending communities | 1.2 x 10^0^ |
| c_SA_ | Multiplier on infectiousness for active TB in remaining South African communities | 1.0 x 10^0^ |

Table S4. Population mixing-related parameters in the individual-based model. In general, *p*_a-b_ represents the fraction of time that a resident of area *a* spends in area *b*.

## Longer-term migration: Labor-sending to mining residency status change

### The mining group was populated by individuals from the labor-sending group. In the model, 31% of individuals born into the labor-sending group were made eligible for a change in residency status, thereby representing the majority of males in the labor-sending population. This transition occurred after age 18 and was specified by a probability density function corresponding to a cumulative distribution function of 80%, 96%, 98%, and 99% at ages 20, 31, 41, or 50, respectively. Mining group individuals were given a subsequent change in status to represent retirement from mining employment. This occurred via a single exponentially distributed waiting time with with mean 31.75 years.

## Individual-based model calibration methods

Values of model parameters were inferred from fitting the dynamic model simultaneously to country-level annual disease incidence and mortality estimates (2000-2014) as well as disease incidence estimates from mining communities in 2008 from the Thibela TB study. For each data measurement $x_{t}^{j}$, where *j* and *t* represent the type of data and time, respectively, we assume errors were normally distributed. The likelihood of the dynamic model input parameter vector $ϴ$, $L_{t}^{j}(ϴ)$ was then defined as

$L_{t}^{j}\left( ϴ \right)\propto e^{-\frac{\left( x_{t}^{j}-f_{t}^{j}\left( ϴ,s \right) \right)^{2}}{2{}^{2}}}$

or more conveniently in terms of log-likelihood (up to an additive constant) as

$$\log\left( L_{t}^{j}\left( ϴ \right) \right)= -\frac{\left( x_{t}^{j}-f_{t}^{j}\left( ϴ,s \right) \right)^{2}}{2{}_{t}^{j}{}^{2}}$$

Here ${}^{2}$ represents the variance associated with the uncertainty in the data $x_{t}^{j}$, and *s* represents the random number seed used to initialize the dynamic model such that $f_{t}^{j}(ϴ,s)$ represents one statistically correct trajectory of the dynamic model with input parameter $ϴ$.

We sought to give the mining incidence data comparable importance in our calibration to country-level data and simultaneously account for mining incidence data and overall country time trends; therefore, we assigned a calibration weight $cw_{j}$ to each type of data in the calibration. Assuming data points were independent and incorporating the calibration weights, we arrived at the overall log-likelihood function, which could be expressed up to an additive constant as:

$$\log\left( L\left( ϴ \right) \right)= - cw_{j}\frac{\left( x_{t}^{j}-f_{t}^{j}\left( ϴ,s \right) \right)^{2}}{2{}_{t}^{j}{}^{2}}$$

We chose weights for country-level incidence, country-level mortality, and mining incidence as 2/15, 2/15, and 1, respectively. Because we had 15 time points for country-level mortality and incidence from 2000-2014 and one measurement of mining incidence at 2008, we divided the data into two periods (2000-2007 and 2008-2014) and gave country-level incidence and mortality and mining incidence over each period equal contributions in terms of geometric-mean likelihood over the time periods.

The calibration procedure is carried out in an iterative manner using Incremental Mixture Importance Sampling (IMIS) using the likelihood function. We conducted seven iterations, where each iteration consisted of 200 sample points in parameter space for a total of 1400 points. In the first iteration 200 points in parameter space were chosen independently for each parameter via Latin Hypercube Sampling from prior distributions on the parameters for relative mine transmission rate and susceptibility to re-infection. We assumed the prior distribution for susceptibility to re-infection to be uniform, Susc_p ~ U (0, 1). Similarly we assumed the relative mine transmission rate prior distribution to be U (1, 5), representing transmission ranging from one to five times the overall country-level rate. We denoted $p(ϴ)$ as the prior probability density and $ϴ_{i}^{j}, i=1\ldots n$ as all of the sample points up to and including the *j*^th^ iteration. We computed the importance weights for the first iteration of the algorithm as:

$$w_{i}^{(1)}=\frac{L\left( ϴ_{i}^{1} \right)}{\sum{L(ϴ}_{i}^{1})}$$

Points for the next iteration were chosen by constructing a multivariate normal distribution centered at the value $ϴ_{i*}^{1}$ which maximized$w^{1}$ with a weighted covariance matrix constructed from the other points in the sample.

We denoted this multivariate normal distribution as $H_{1}$ then chose 200 new points from this distribution and computed their likelihood under the dynamic model. We then constructed the importance weights for all of the points up to and including the second generation as:

$$w_{i}^{(2)}\alpha L(ϴ_{i})\frac{p\left( ϴ_{i} \right)}{q^{1}(ϴ_{i})}$$

where *q* is the density of a mixture distribution consisting of the prior distribution and the multivariate normal distribution $H_{1}$, and the mixture was weighted by the number of points in each iteration in proportion to the total. In this case since we had an equal number of points in each iteration

$$q^{1}=\frac{1}{2}p+\frac{1}{2}H_{1}$$

Points in subsequent iterations were chosen in the same manner. For the *j*th generation we defined a multivariate normal distribution centered at the input parameter with the maximum importance weight $w_{i*}^{(j-1)}$ with a weighted covariance matrix constructed from the 200 points closest in terms of Mahalanobis distance with respect to the prior distribution $ϴ_{i*}$. New importance weights were then computed with $q^{j}=\frac{1}{j}p+\frac{1}{j}\sum H_{i}$. In the seventh and final iteration, we computed the sample posterior distribution of $ϴ$by sampling without replacement from the importance weights $w_{i}^{(7)}$ corresponding to each $ϴ_{i}$.

## Individual-based model calibration results

The estimated joint posterior probability density for the calibrated model parameters, $\beta_{mines}^{rel}$ and $su_{re}$ is shown in Figure S2A. Marginal densities for the parameters are shown in Figure S2B and S2C. The distributions are unimodal and strongly peaked, with a maximum *a posteri* value located at $\beta_{mines}^{rel}=2.75$ and $u_{re}=0.53$. Because protection from future disease in previously exposed individuals in the model works through prevention of re-infection, the estimate of protection from re-infection was similar to estimates of BCG protection against active disease of 0.58 (CI95: 0.35-1.01) (Mantgani et al. 2014).

Posterior predictive distributions for disease incidence and mortality are shown at the country level (Figures S3A, S3B) and specifically in the mining population (Figure S3C, S3D). Note that country level values were computed as a population-weighted average of the four groups in the model. The distributions recapitulated the time trends in both mortality and incidence and were within the bounds of the WHO estimates indicated by the error bars. Similarly predicted incidence in the mining community recapitulated the 2008 Thibela TB estimates. In addition, annual incidence in the early 2000s ranging from ~2 000 to 6 000 per 100 000 was consistent with other mining study populations from this period. Mining community TB disease mortality was predicted to be ~1% per annum during this period which was also consistent with mine records showing overall mortality of ~1% and 4% sent home due to illness.

## Individual-based model reproduction of Thibela TB study

As an additional test of the individual-based model, we simulated the Thibela TB study conditions and predicted the impact of a program administering a 9-month course of IPT similar to that from the Thibela TB study between 2006 and 2010. For comparison we simulated rollout to a cohort of individuals with LTBI beginning in 2008 with the intervention adopted over 18 months, i.e., rolling enrollment, assuming 60% population coverage, complete prevention of reactivation on IPT, and either 0% or 30% probability of clearance of LTBI on IPT, where the latter scenario of 30% clearance was similar to the value found by Sumner et al. [[11]](https://paperpile.com/c/5kxPgO/qy3Fr). In both cases we observe an initial decline in TB incidence which rebounds quickly to the level of baseline incidence (Figure S4A). Two years after the introduction of the intervention, the incidence reduction was consistent with that estimated from the Thibela TB trial [[4]](https://paperpile.com/c/5kxPgO/FVzWj). Differences between the two cases were observed with respect to LTBI prevalence, but these differences were largely eliminated after intervention cessation (Figure S4B).

## Computing population attributable fraction of incidence

We computed the mean incidence measured over a time window $[t_{o}, t_{0}+h_{1}]$ attributable to transmission from a given group occurring over the time window $[t_{0}-h_{0}, t_{o}+h_{1}]$. Here we allowed for the transmission time window to extend further into the past as compared to the incidence window to account for the delay from infection to disease.

Given a point in our epidemiological input parameter space $ϴ$ and an input random number seed for the stochastic model *s*, we denoted $I_{\left[ t_{0}, t_{0}+h_{1} \right]}(ϴ,s)$ to be the cumulative disease incidence in terms of cases of the model over the time period $[t_{o}, t_{0}+h_{1}]$. Similarly, for the counterfactual model where we had artificially removed transmission from group *G* beginning at time $t_{0}-h_{0}$ we denoted the corresponding cumulative incidence to be$I_{\left[ t_{0}, t_{0}+h_{1} \right]}^{G,h_{0}}(ϴ,s)$. For the stochastic simulation it followed that the attributable fraction could be computed as

$Paf_{G}\left( ϴ,s \right)=\frac{{I_{\left[ t_{0}, t_{0}+h_{1} \right]}\left( ϴ,s \right)-I}_{\left[ t_{0}, t_{0}+h_{1} \right]}^{G,h_{0}}\left( ϴ,s \right)}{I_{\left[ t_{0}, t_{0}+h_{1} \right]}\left( ϴ,s \right)}$

We then estimated the mean attributable fraction as the sample mean over a large set of independent identically distributed stochastic simulations defined by the set of input random number seeds $\left\{ s_{i} \right\} i=1\ldots N$

$Paf(ϴ)$ = <$Paf_{G}\left( ϴ,s \right)>_{s}$

and computed the associated confidence intervals. Analogously we also estimated the fraction of incidence from recent transmission, i.e., incidence from any transmission occurring over the time window $[t_{0}-h_{0}, t_{o}+h_{1}]$ attributable to a group *G* as

$Paf_{G}^{Recent}\left( ϴ,s \right)=\frac{Paf_{G}\left( ϴ,s \right)}{Paf_{All}\left( ϴ,s \right)}$ , $Paf_{G}^{Recent}\left( ϴ \right)= <Paf_{G}^{Recent}\left( ϴ,s \right)>_{s}$

where *All* denotes all groups in the model. Table 3 provides estimates and confidence limits of incidence fractions attributable to mining, $Paf_{Mines}$and $Paf_{Mines}^{Recent}$, for the estimated most likely input parameters. Here we defined $t_{0}=2014, h_{0}=2, h_{1}=5$ years corresponding to a five-year incidence window beginning in 2014 and recent transmission as occurring after 2012. The sample mean and confidence limits were based on N=200 stochastic simulations.

## Sensitivity analysis

To test the robustness of the results to assumptions about the infectiousness in each setting, we performed a one-way sensitivity analysis by varying the setting-specific multipliers on *Mtb* transmission (c_M_, c_PM_, c_LS_, c_SA_, Table S4) one at a time +30% or -30% of their calibrated values. The steps to calculate the contribution of *Mtb* transmission in the mines was then repeated (as described in Methods, "Individual-based model calibration and application").

# References

[1. Huynh GH, Klein DJ, Chin DP, Wagner BG, Eckhoff PA, Liu R, et al. Tuberculosis control strategies to reach the 2035 global targets in China: the role of changing demographics and reactivation disease. BMC Med. 2015;13:88.](http://paperpile.com/b/5kxPgO/Kgtgz)

[2. Houben R. Feasibility of achieving the 2025 WHO Global TB Targets in South Africa, China, and India: A combined analysis of 11 models. Lancet Global Health. 2016;](http://paperpile.com/b/5kxPgO/NkfKo)

[3. Kon ZR, Lackan N. Ethnic disparities in access to care in post-apartheid South Africa. Am. J. Public Health. 2008;98:2272–7.](http://paperpile.com/b/5kxPgO/DO3Xq)

[4. Churchyard GJ, Fielding KL, Lewis JJ, Coetzee L, Corbett EL, Godfrey-Faussett P, et al. A trial of mass isoniazid preventive therapy for tuberculosis control. N. Engl. J. Med. 2014;370:301–10.](http://paperpile.com/b/5kxPgO/FVzWj)

[5. Bershteyn A, Klein DJ, Wenger E, Eckhoff PA. Description of the EMOD-HIV Model v0.7 [Internet]. arXiv [q-bio.QM]. 2012. Available from:](http://paperpile.com/b/5kxPgO/kLXAT) <http://arxiv.org/abs/1206.3720>

[6. Shisana O, Rehle T, Simbayi LC, Zuma K, Jooste S, Zungu N, et al. South African national HIV prevalence, incidence and behaviour survey, 2012. HSRC press; 2014; Available from:](http://paperpile.com/b/5kxPgO/giM0q) <http://repository.hsrc.ac.za/handle/20.500.11910/2490>

[7. Pronyk RM, Makhubele MB, Hargreaves JR, Tollman SM, Hausler HP. Assessing health seeking behaviour among tuberculosis patients in rural South Africa. Int. J. Tuberc. Lung Dis. 2001;5:619–27.](http://paperpile.com/b/5kxPgO/Tj15C)

[8. Yimer S, Holm-Hansen C, Yimaldu T, Bjune G. Health care seeking among pulmonary tuberculosis suspects and patients in rural Ethiopia: a community-based study. BMC Public Health. 2009;9:454.](http://paperpile.com/b/5kxPgO/u82i9)

[9. Maynard-Smith L, Larke N, Peters JA, Lawn SD. Diagnostic accuracy of the Xpert MTB/RIF assay for extrapulmonary and pulmonary tuberculosis when testing non-respiratory samples: a systematic review. BMC Infect. Dis. 2014;14:709.](http://paperpile.com/b/5kxPgO/5v3KX)

[10. Organization WH, Others. Global tuberculosis report 2016. World Health Organization; 2016; Available from:](http://paperpile.com/b/5kxPgO/VwUtH) <http://apps.who.int/iris/bitstream/10665/250441/1/9789241565394-eng.pdf>

[11. Sumner T, Houben RMGJ, Rangaka MX, Maartens G, Boulle A, Wilkinson RJ, et al. Post-treatment effect of isoniazid preventive therapy on tuberculosis incidence in HIV-infected individuals on antiretroviral therapy. AIDS. 2016;30:1279–86.](http://paperpile.com/b/5kxPgO/qy3Fr)

[12. World Health Organization. South Africa statistics summary (2002 - present) [Internet]. Global Health Observatory. [cited 2017 Sep 11]. Available from:](http://paperpile.com/b/5kxPgO/dHHjz) <http://apps.who.int/gho/data/node.country.country-ZAF?lang=en>

[13. Stats SA. Census 2011. Statistics South Africa, Pretoria. 2011;](http://paperpile.com/b/5kxPgO/qCqt5)

# Results

## Monte Carlo simulations of static risk model: FOI

|  | ***From Mining residents*** | ***From Peri-mining residents*** | ***From Labor-sending residents*** | ***From Other SA residents*** | ***From all residents*** |
| --- | --- | --- | --- | --- | --- |
| ***Among Mining residents*** | (1.52, 2.48) x 10^-1^  (92.4%, 95.1%) | (4.40, 4.43) x 10^-3^  (1.7%, 2.7%) | (6.92, 7.04) x 10^-3^  (2.7%, 4.2%) | (1.25, 1.39) x 10^-3^  (0.5%, 0.8%) | (1.64, 2.61) x 10^-1^  (100%) |
| ***Among Peri-mining residents*** | (2.48, 2.49) x 10^-3^  (5.8%, 5.8%) | (3.90, 3.90) x 10^-2^  (91.4%, 91.5%) | (5.73, 5.73) x 10^-4^  (1.3%, 1.3%) | (5.93, 5.93) x 10^-4^  (1.4%, 1.4%) | (4.27, 4.27) x 10^-2^  (100%) |
| ***Among Labor-sending residents*** | (2.07,2.10) x 10^-3^  (3.5%, 3.6%) | (3.04, 3.04) x 10^-4^  (0.5%, 0.5%) | (5.52, 5.52) x 10^-2^  (94.7%, 94.7%) | (6.88, 6.88) x 10^-4^  (1.1%, 1.1%) | (5.83, 5.83) x 10^-2^  (100%) |
| ***Among Other SA residents*** | (3.70, 4.07) x 10^-5^  (0.1%, 0.1%) | (3.15, 3.15) x 10^-4^  (0.9%, 0.9%) | (6.80, 6.80) x 10^-5^  (0.2%, 0.2%) | (3.47, 3.47) x 10^-2^  (98.8%, 98.8%) | (3.51, 3.51) x 10^-2^  (100%) |

Table S5. Force of infection (per-susceptible rate of infection) attributable to each residency group, as per-annum rate and as a percentage of total from all groups, as CI95 from 1000 Monte Carlo simulations. Differences of <0.05% in CI95 are indicated by identical values in CI95.

## Monte Carlo simulations of static risk model: Incidence

|  | ***Model*** | ***Published value*** |
| --- | --- | --- |
| ***Among Mining residents*** | 2963 (2208, 3858) | 2957 |
| ***Among Peri-mining residents*** | 1134 (1064, 1200) | N/A |
| ***Among Labor-sending residents*** | 1402 (1402, 1402) | N/A |
| ***Among Other SA residents*** | 931 (931, 932) | N/A |
| ***Among all residents*** | 989 (980, 1000) | 977 (717, 1276) |

## Table S6. Incidence (active TB cases per 100 000 population per annum) mean and CI95 output by the static risk model as compared to published values for mining areas from Thibela TB study control clusters [[4]](https://paperpile.com/c/5kxPgO/FVzWj) and all of South Africa for 2008 [[10,12]](https://paperpile.com/c/5kxPgO/VwUtH+dHHjz). N/A, not available.

## Monte Carlo simulations of static risk model: New infections

|  | ***From Mining residents*** | ***From Peri-mining residents*** | ***From Labor-sending residents*** | ***From Other SA residents*** | ***From all residents*** |
| --- | --- | --- | --- | --- | --- |
| ***New infections among all SA residents*** | (3.89, 9.10) x 10^4^  (2.6%, 5.8%) | (6.82, 8.68) x 10^4^  (4.5%, 5.5%) | (1.37, 1.39) x 10^5^  (8.8%, 9.1%) | (1.26, 1.26) x 10^6^  (80.0%, 83.7%) | (1.51, 1.58) x 10^6^  (100%) |
| ***Population size of attributable source*** | 4.85 x 10^5^  (0.9%) | 2.14 x 10^6^  (4.1%) | 3.35 x 10^6^  (6.5%) | 4.58 x 10^7^  (88.4%) | 5.18 x 10^6^  (100%) |
| ***Ratio of new infection % : population %*** | (2.77, 6.15) | (1.09, 1.34) | (1.36, 1.41) | (0.90, 0.95) | (1.00) |
| ***Prevalence (est.) in attributable source*** | 1.04 x 10^4^  (2.5%) | 1.84 x 10^4^  (4.4%) | 3.47 x 10^4^  (8.4%) | 3.52 x 10^5^  (84.7%) | 4.15 x 10^5^  (100%) |
| ***Ratio of new infection % : prevalence %*** | (1.04, 2.30) | (1.02, 1.25) | (1.05, 1.09) | (0.95, 0.99) | (1.00) |

Table S7. New infections in all South Africa attributable to each residency group, as number of cases and percentage of total; ratio of percentage of new infections to percentage of total population; and ratio of percentage of new infections to percentage of total prevalence, as CI95 from 1000 Monte Carlo simulations.

##

## Demographics in the individual-based model

| **A**  **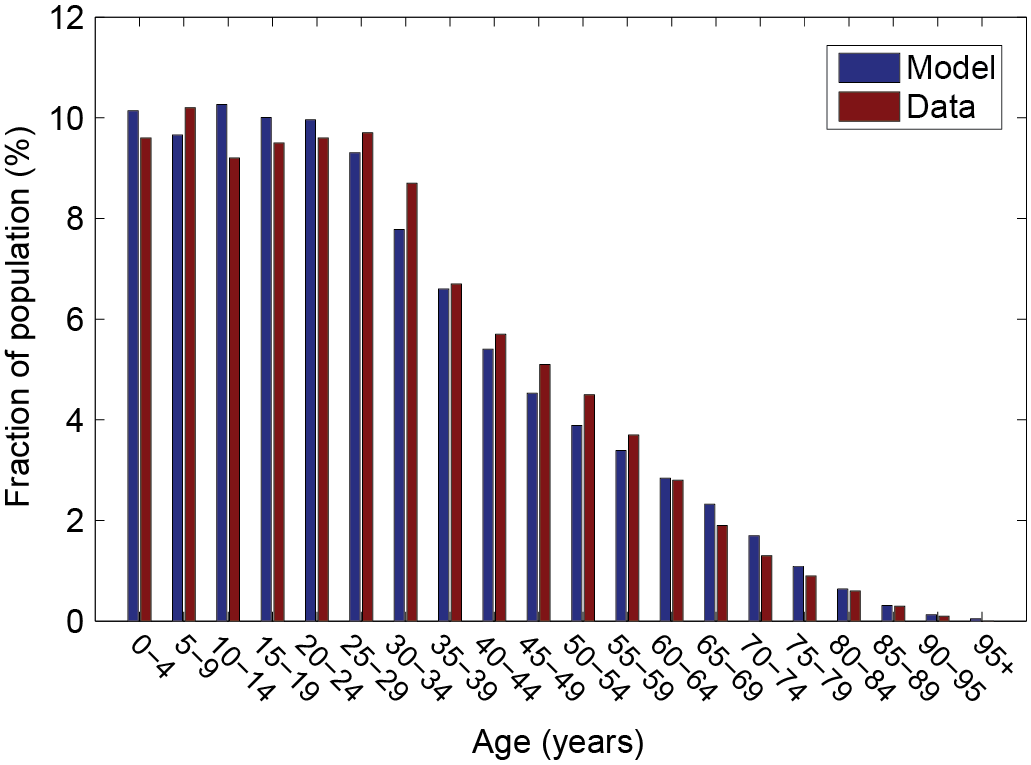** | **B**  **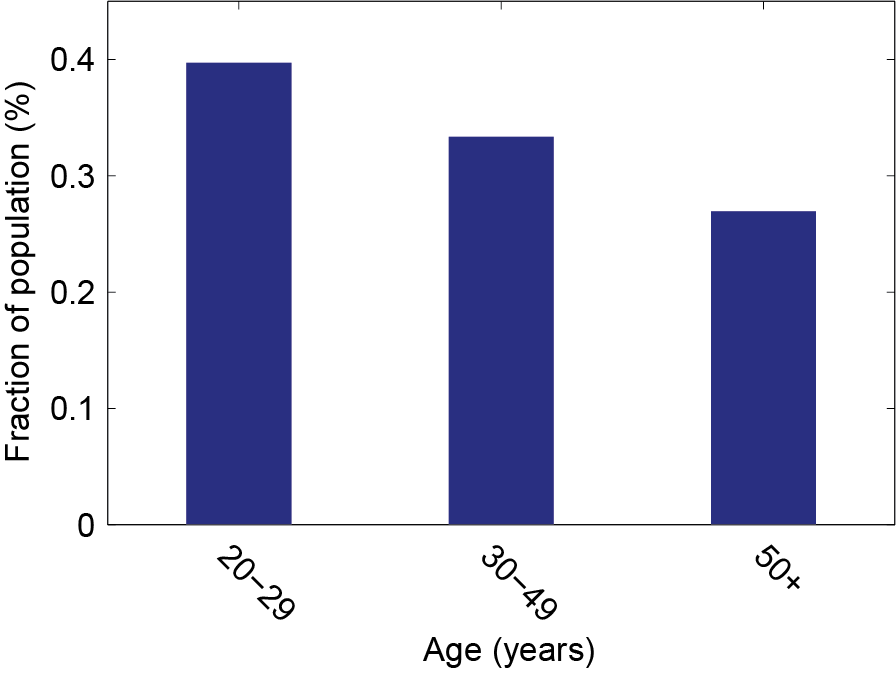** |
| --- | --- |
| **C**  **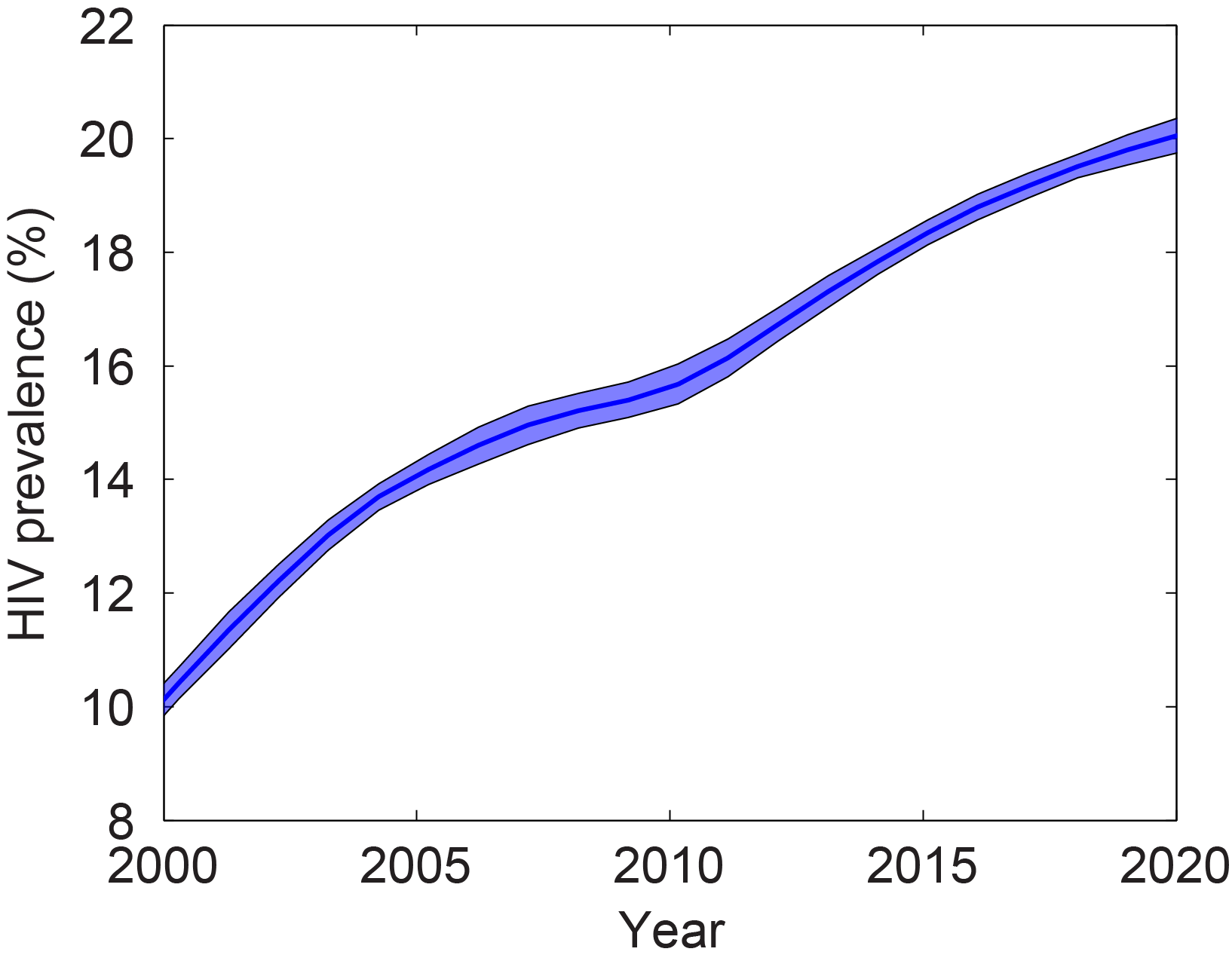** | **D**  **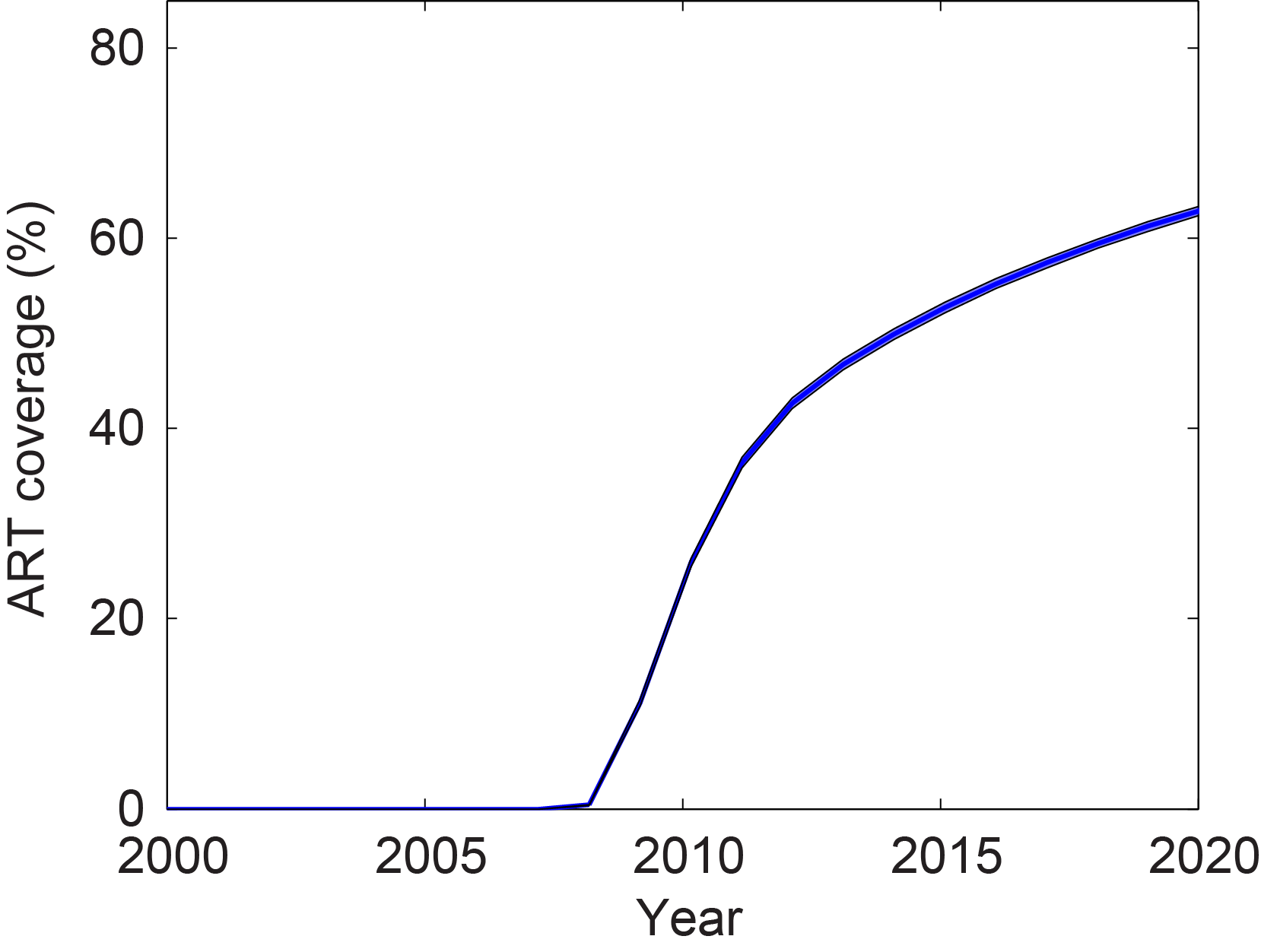** |

## Figure S1. Demographics in the individual-based TB model. (A) Age distribution of individuals (excluding mine workers) in the model (blue) compared to 2011 census data (red) [[13]](https://paperpile.com/c/5kxPgO/qCqt5). (B) Age distribution of mine workers in the model. (C) HIV prevalence in the overall population in the model. (D) ART coverage of HIV-positive individuals in the overall population in the model.

## Calibrated parameters from the individual-based model

| **A**  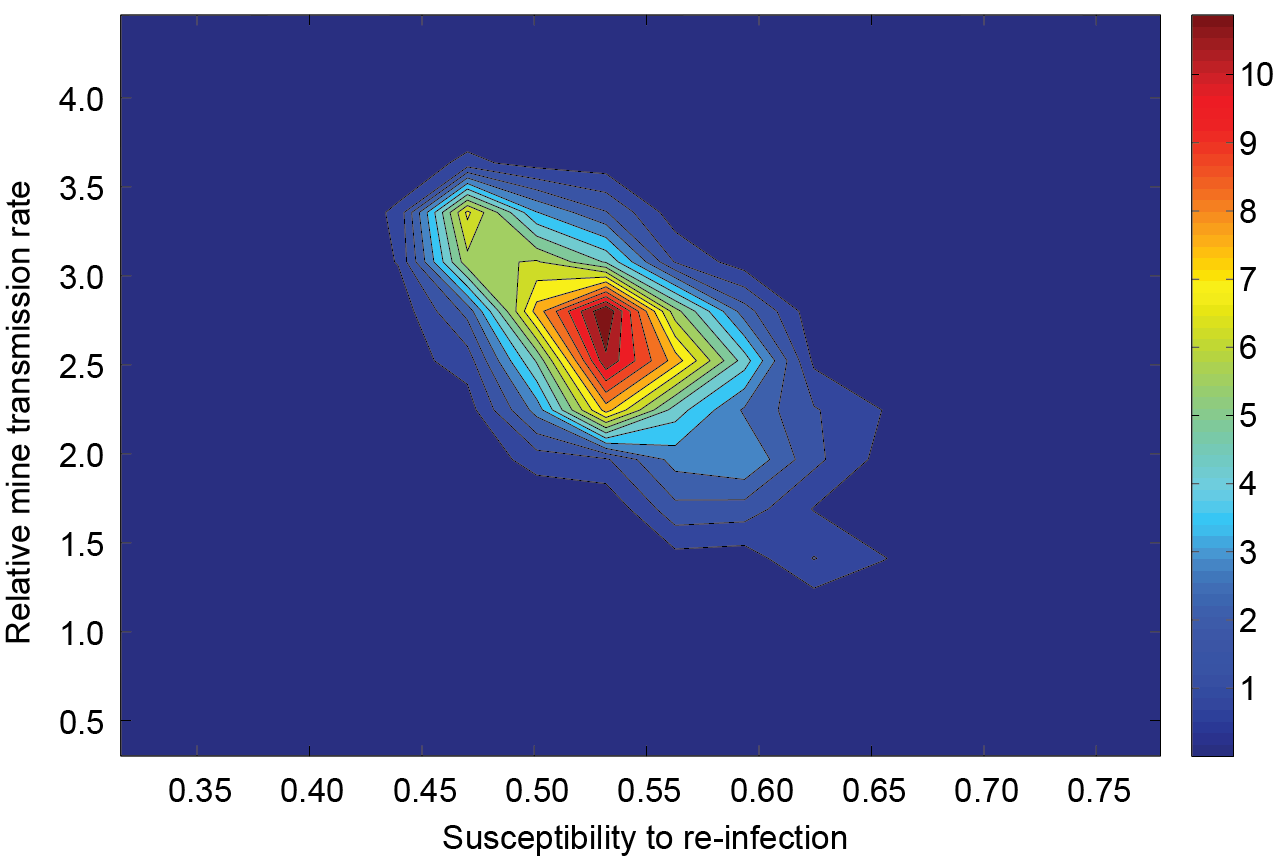 | **B**  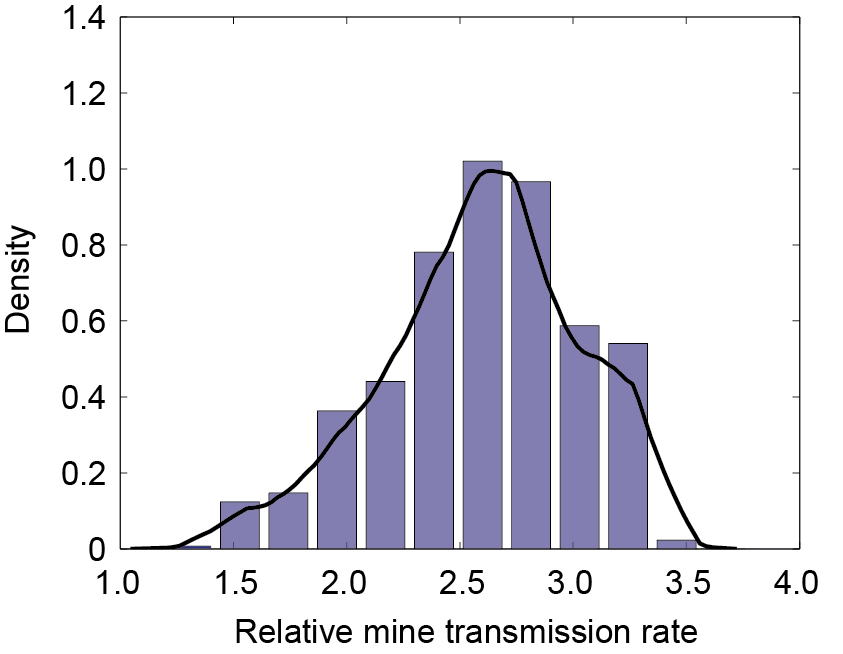 |
| --- | --- |
| **C**  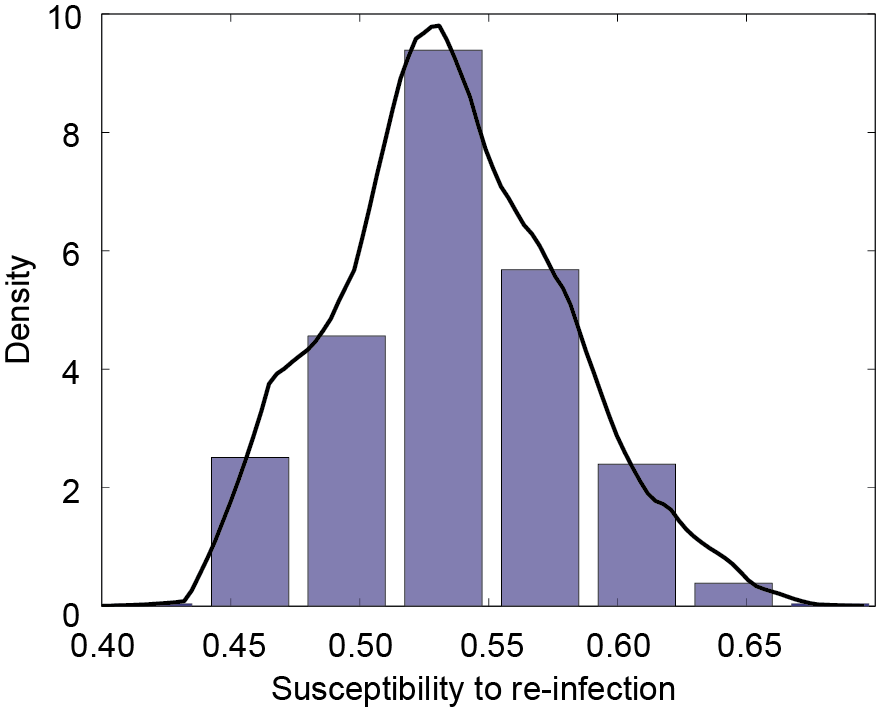 | **D**  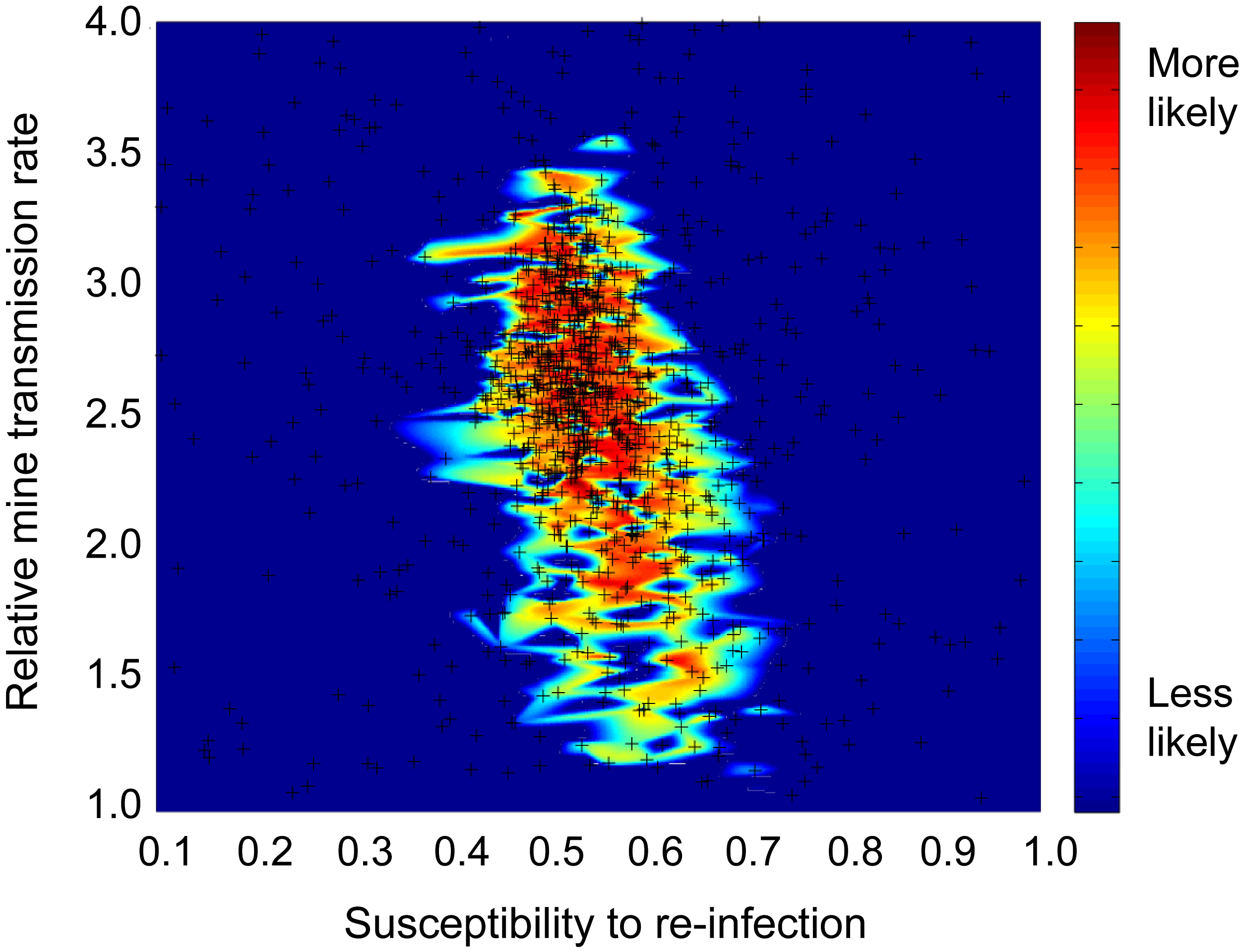 |

## Figure S2. Parameters resulting from model calibration to incidence and mortality data. (A) Estimated joint probability density for the posterior distribution of model input parameters. The posterior density was estimated via IMIS and two-dimensional kernel density smoothing. (B) Marginal posterior probability density of the relative mine transmission rate parameter. (C) Marginal posterior probability density of the susceptibility to reinfection parameter. (D) Log-likelihood for sample parameters points from successive IMIS iterations.

## Calibrated baseline outputs from the individual-based model

| **A**  **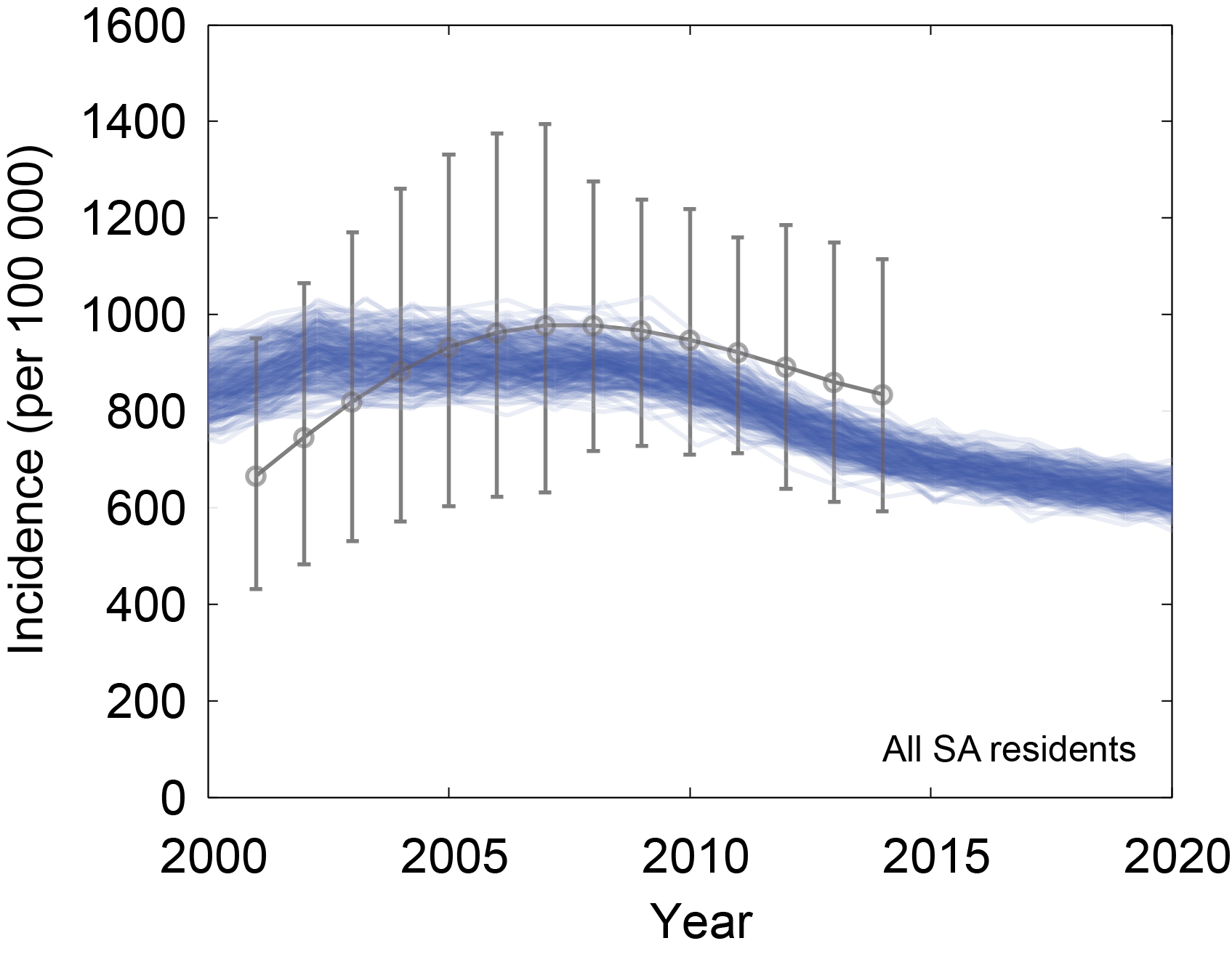** | **B**  **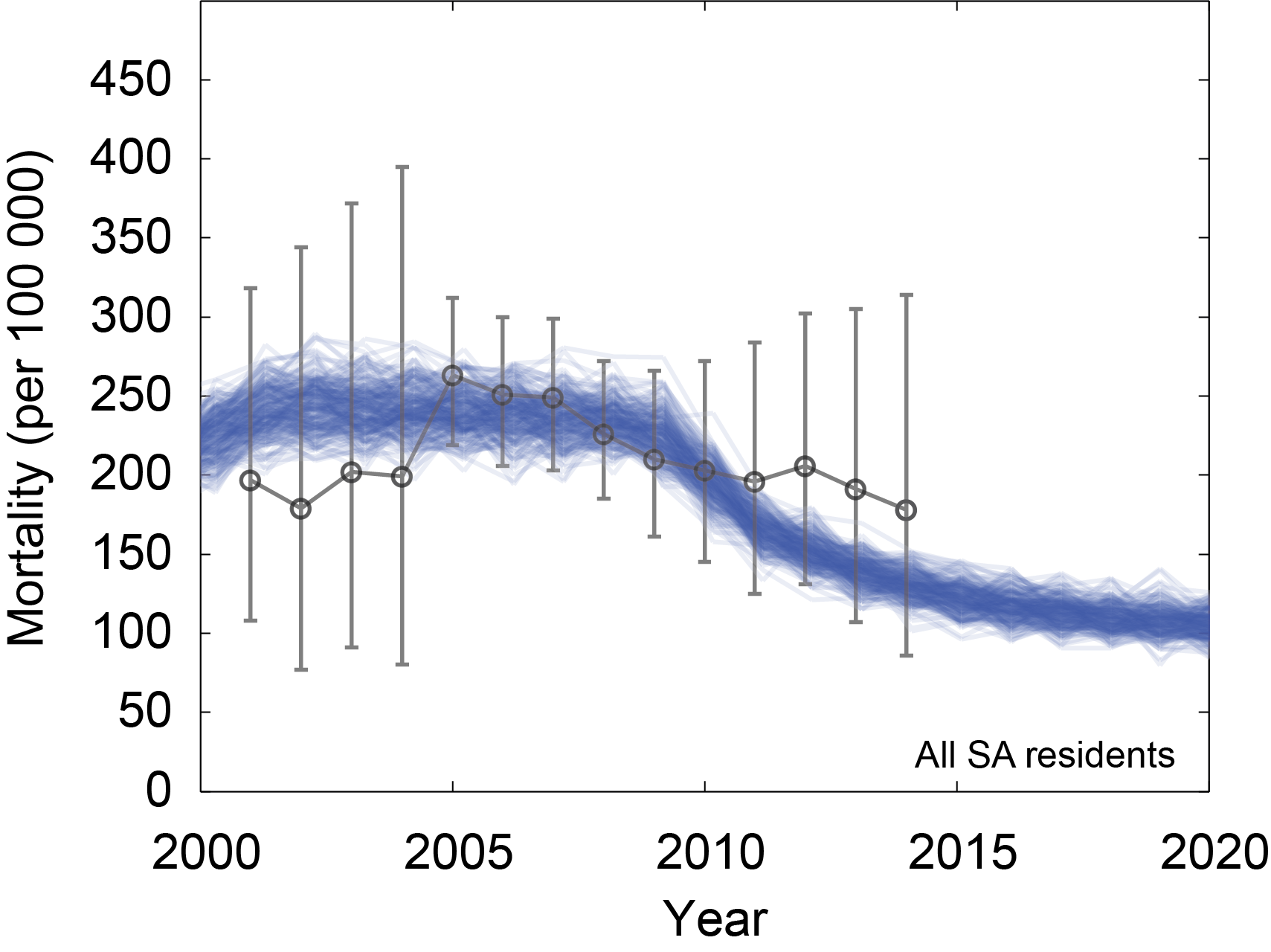** |
| --- | --- |
| **C**  **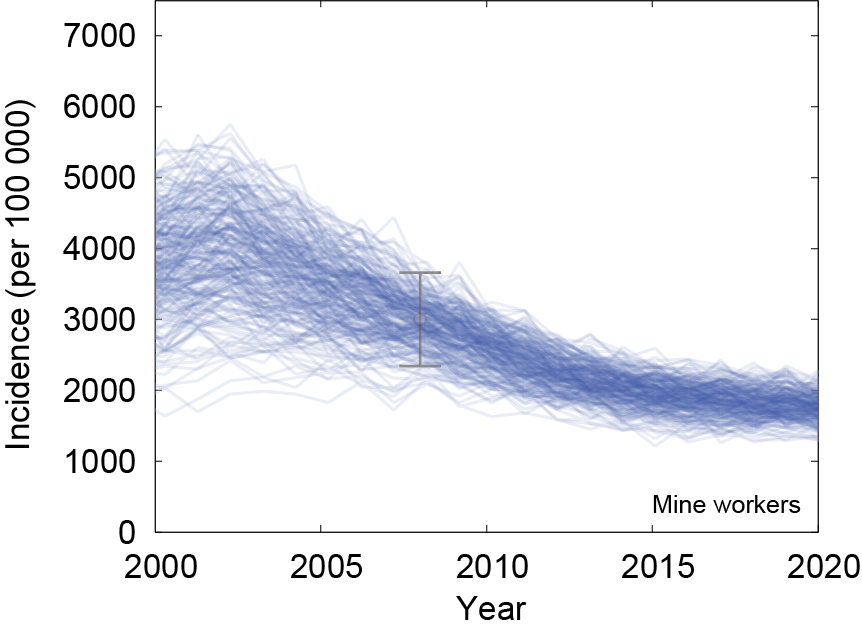** | **D**  **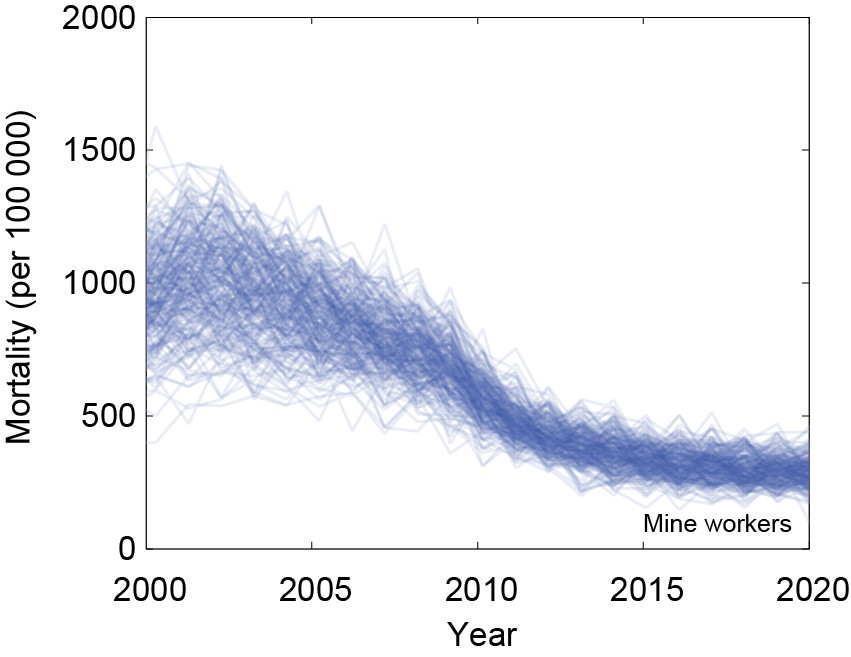** |

Figure S3. Posterior predictive distributions. (A) Country-level TB incidence overlaid with WHO estimates [[10]](https://paperpile.com/c/5kxPgO/VwUtH). (B) Country-level TB mortality overlaid with WHO estimates [[10]](https://paperpile.com/c/5kxPgO/VwUtH). (C) Mine worker TB incidence overlaid with case notification rate from Thibela TB study [[4]](https://paperpile.com/c/5kxPgO/FVzWj). (D) Mine worker TB mortality. Curves are shown with partial transparency such that color intensity is proportional to probability density.

## Simulation of Thibela TB study in the individual-based model

| **A**  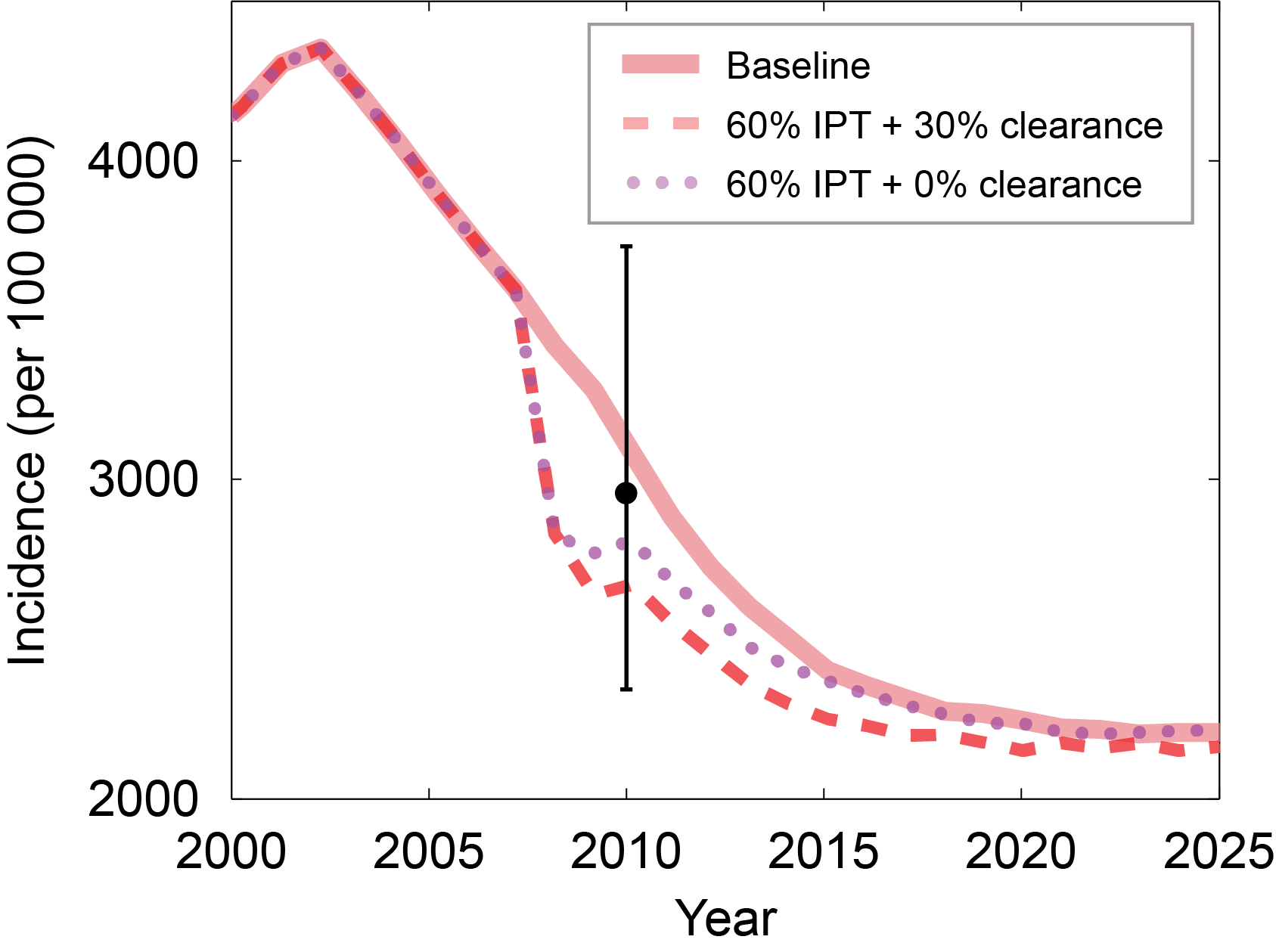 | **B**  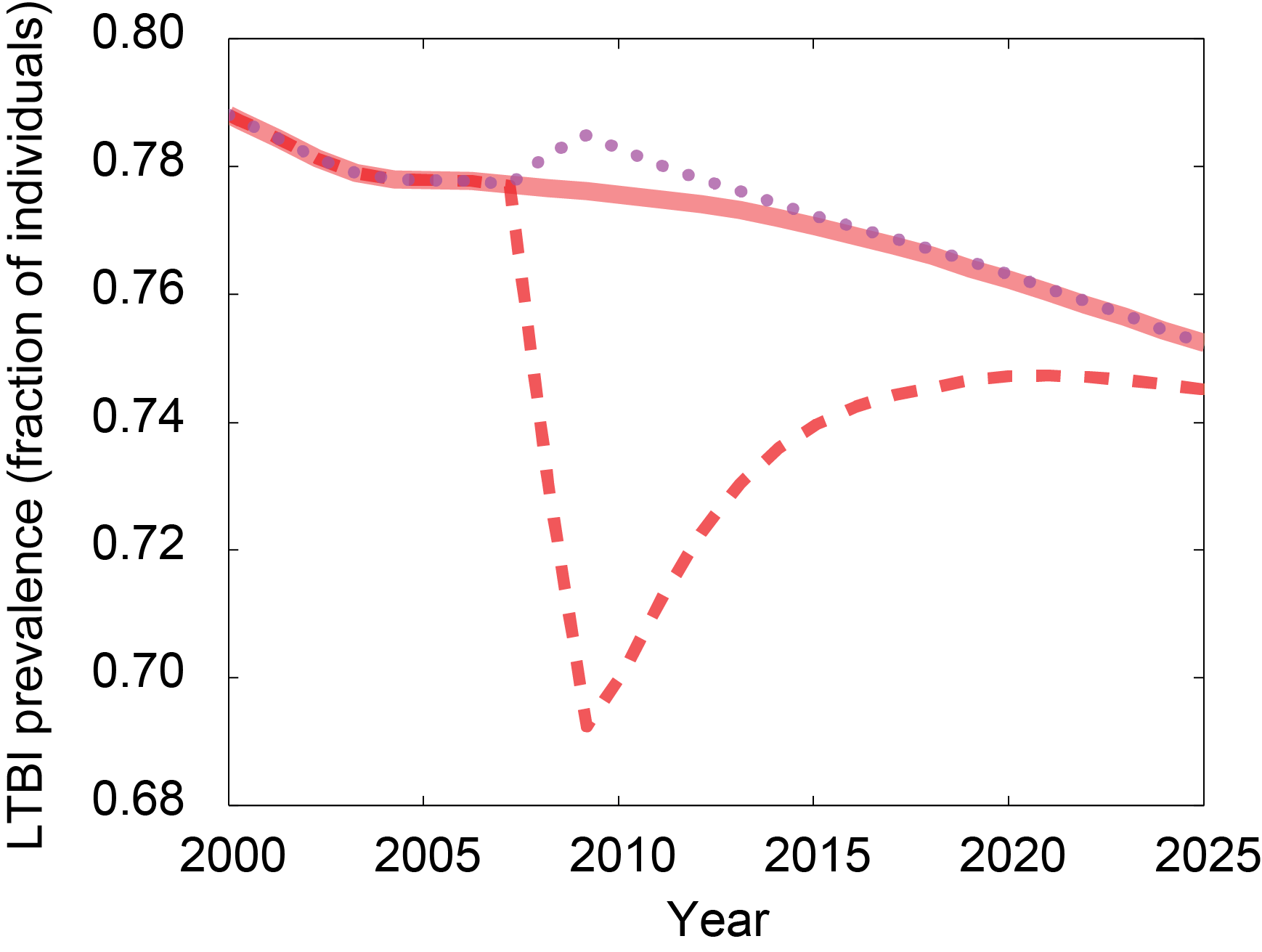 |
| --- | --- |

Figure S4. Simulation of Thibela TB study conditions and predicted impact of population-wide isoniazid preventive therapy (IPT). (A) TB incidence. (B) LTBI prevalence. "60% IPT + 0% clearance" represents 60% coverage of the LTBI population in 2008, similar to the adoption rate in the Thibela TB study, with enrollment over 18 months, prevention of reactivation while receiving IPT, and 0% probability of LTBI clearance. "60% IPT + 30% clearance" represents the same conditions with 30% probability of LTBI clearance. The data point represents the incidence estimate from the Thibela TB study [[4]](https://paperpile.com/c/5kxPgO/FVzWj).

## Sensitivity analysis

| 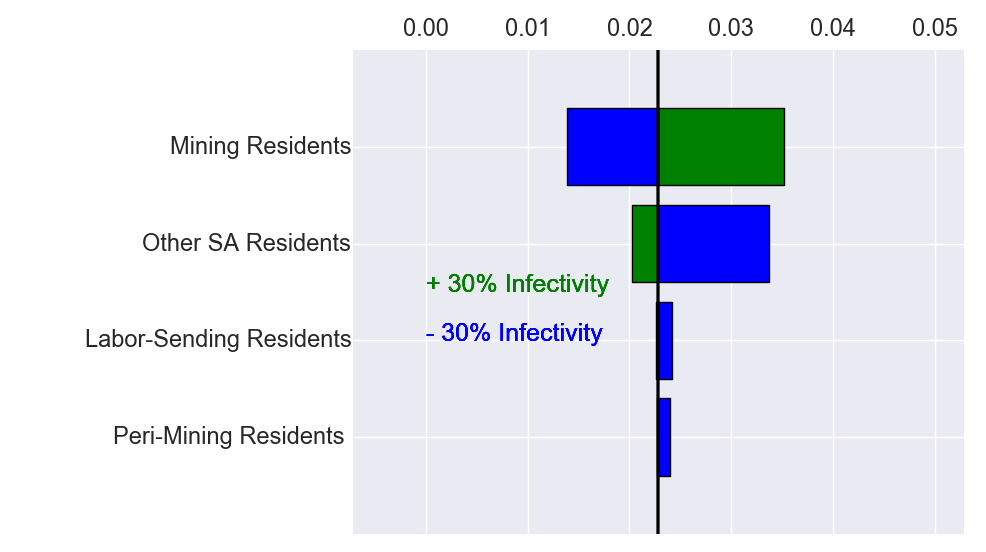 |
| --- |

Figure S5. Sensitivity analysis of the fraction of TB incidence due to recent *Mtb* transmission in the mines. The infectiousness in each group was varied one at a time +/- 30%, and the fraction of TB incidence measured via the counterfactual method.
